# Identification of pharmacokinetic markers for safflower injection using a combination of system pharmacology, multicomponent pharmacokinetics, and quantitative proteomics study

**DOI:** 10.3389/fphar.2022.1062026

**Published:** 2022-11-23

**Authors:** Peiying Shi, Yijun Ruan, Chenhui Zhong, Linglin Teng, Liyuan Ke, Hong Yao

**Affiliations:** ^1^ Department of Traditional Chinese Medicine Resource and Bee Products, College of Animal Sciences (College of Bee Science), Fujian Agriculture and Forestry University, Fuzhou, China; ^2^ State and Local Joint Engineering Laboratory of Natural Biotoxins, Fujian Agriculture and Forestry University, Fuzhou, China; ^3^ Department of Pharmaceutical Analysis, School of Pharmacy, Fujian Medical University, Fuzhou, China; ^4^ Fujian Key Laboratory of Drug Target Discovery and Structural and Functional Research, Fujian Medical University, Fuzhou, China

**Keywords:** PK marker, safflower injection, multicomponent pharmacokinetics, system pharmacology, quantitative proteomics, hydroxysafflor yellow A

## Abstract

Safflower injection (SI), a water-extract preparation from safflower (*Carthamus tinctorius* L.), has been widely used for the treatment of cardio-cerebrovascular diseases. This work aims to develop an approach for identifying PK markers of cardiovascular herbal medicines using SI as a case study. Firstly, qualitative and quantitative analyses were performed to reveal ingredients of the preparation *via* HPLC-MS. Subsequently, multiple PK ingredients and integrated PK investigations were carried out to ascertain ingredients with favorable PK properties (e.g., easily detected at conventional PK time points and high system exposure) for the whole preparation. Next, ingredients against cardiovascular diseases (CVDs) in the preparation were predicted with target fishing and system pharmacology studies. Finally, ingredients with favorable PK properties, satisfactory PK representativeness for the preparation, and high relevance to CVDs were considered as potential PK markers. Their therapeutic effect was further evaluated using the H_2_O_2_-induced H9c2 cardiomyocyte-injured model and a proteomics study to identify objective PK markers. As results, it disclosed that SI mainly contains 11 ingredients. Among them, five ingredients, namely, hydroxysafflor yellow A (HSYA), syringin (SYR), *p*-coumaric acid (*p*-CA), scutellarin (SCU), and *p*-hydroxybenzaldehyde (*p*-HBA), showed favorable PK properties. HSYA, SYR, and rutin (RU) were predicted to show high relevance to CVDs and screened as potential PK markers. However, only HSYA and SYR were confirmed as therapeutic ingredients against CVDs. Combined with these findings, only HSYA demonstrated satisfactory representativeness on PK properties and therapeutic effects of multiple ingredients of the preparation, thereby indicating that HSYA is a potential PK marker for the SI. The results of this study can provide a reference for the characterization of PK markers for traditional Chinese medicines.

## 1 Introduction

Traditional Chinese medicines (TCMs) have been used in clinical practice for thousands of years in Asian countries. TCMs, especially their preparations for the treatment of cardio-cerebrovascular and respiratory tract infection diseases, still play an important role in Chinese medication and health system due to their generally accepted effectiveness and safety, although the Western medicine system typically dominates the treatment of these diseases in modernized metropolitans. However, till this day, the dosage regimen design for all TCM preparations still mainly relies on ancient practical experience, which lacks the support of modern scientific experiments (Yao et al., 2017; Yan et al., 2018; Shi et al., 2020).

Pharmacokinetic (PK) studies investigate the disposition of drugs in the body by profiling the alternation of drug concentration over time in the circulation, tissues, and/or organs and using mathematical principles and methods to decipher the *in vivo* dynamic alternation law of drugs to ensure that the process of absorption, distribution, metabolism, and excretion can be conveniently understood (Shi et al., 2018; Xu et al., 2021). A series of PK parameters, such as peak concentration (C_max_), biological half-life (T_1/2_), peak time (T_max_), apparent distribution volume (Vd), clearance rate (CL), and area under the drug concentration and time curve (AUC), can be obtained and used to guide the drug dosage regimen design in clinics in the PK investigation for a single-compound western drug. The technology of HPLC-MS has been adopted in the PK study of TCMs based on its high sensitivity and selectivity (Liu et al., 2017). And the results of PK studies on TCM preparations combined with principles and methods of Western medicine PK research can also provide scientific guidance on the dosage regimen design to contribute to the inheritance and innovation of TCMs (Li et al., 2015; Shi et al., 2018). However, TCM preparations often contain complex components. And HPLC-MS has been applied in qualitative and quantitative analysis for metabolites of plants (Liu et al., 2017; Song et al., 2021). PK investigations of multiple-component TCM are very challenging under this condition because we rarely have idea about which one or some ingredients’ PK profiles could reflect the overall *in vivo* process of one TCM studied.

Feasible PK investigation strategies or methods for complex TCM products, such as, PK marker identification and integrated PK investigation, have been extensively investigated (Shi et al., 2018; Chen et al., 2022). Among these studies, PK marker identification is considered a promising method for exploring the overall process of a complex TCM *in vivo*. Active ingredients with dominant contents and favorable PK properties (e.g., appropriate elimination half-life and remarkable dose-dependent systemic exposure) can be suitable PK markers for a certain TCM product (Li et al., 2008; Li, 2017). Scientists aim to explore characteristic compounds as representative PK markers because they can represent therapeutic effects of the TCM preparation *in vivo*. However, the complexity of the component type and quantity of a TCM increases the difficulty in assessing the therapeutic effects of the TCM (Hao et al., 2009; Li et al., 2015; Shi et al., 2018). Therefore, establishing efficient methods or strategies for screening multiple active components of TCMs is the key to solving the problem of PK marker identification of the TCM.

Safflower injection (SI), a water-extract preparation from safflower (*Carthamus tinctorius* L.), has been widely used for the treatment of cardio-cerebrovascular diseases, such as coronary heart disease (Lin, 2012), acute coronary syndromes (Lu et al., 2021), and acute cerebral infarction (Zhang, 2020). Its main components include quinochalcones, flavonoids, phenylpropanoids, nucleosides, organic acids, and other compounds (Zhao et al., 2014; Fan et al., 2019). Its adverse drug reactions have gradually increased with the increase of clinical use, and the most serious one is allergic shock (Sun et al., 2013). Single overdose administration is a risk factor of adverse drug reactions/events caused by SI based on a nested case control study (Jiao et al., 2018). Accordingly, understanding the disposition time course of multiple SI ingredients *in vivo* is crucial to help formulate a rational dosage regimen of SI in clinics. Notably, explorations on screening multiple active components of SI and PK studies of SI only monitoring one ingredient, such as hydroxysafflor yellow A (HSYA) in rabbit plasma after intravenously (*i.v.*) dosing the preparation (Wang and Liang, 2011) with HPLC, are limited. However, the use of HSYA as a representative PK marker for presenting the *in vivo* process of the whole preparation remains scientifically unverified. Other main compounds in SI, such as syringin (SYR), *p*-coumaric acid (*p*-CA), and scutellarin (SCU) (Zhao et al., 2014), must also be considered in the PK study for identifying a certain ingredient (e.g., HSYA) as a PK marker.

**SCHEME 1:**
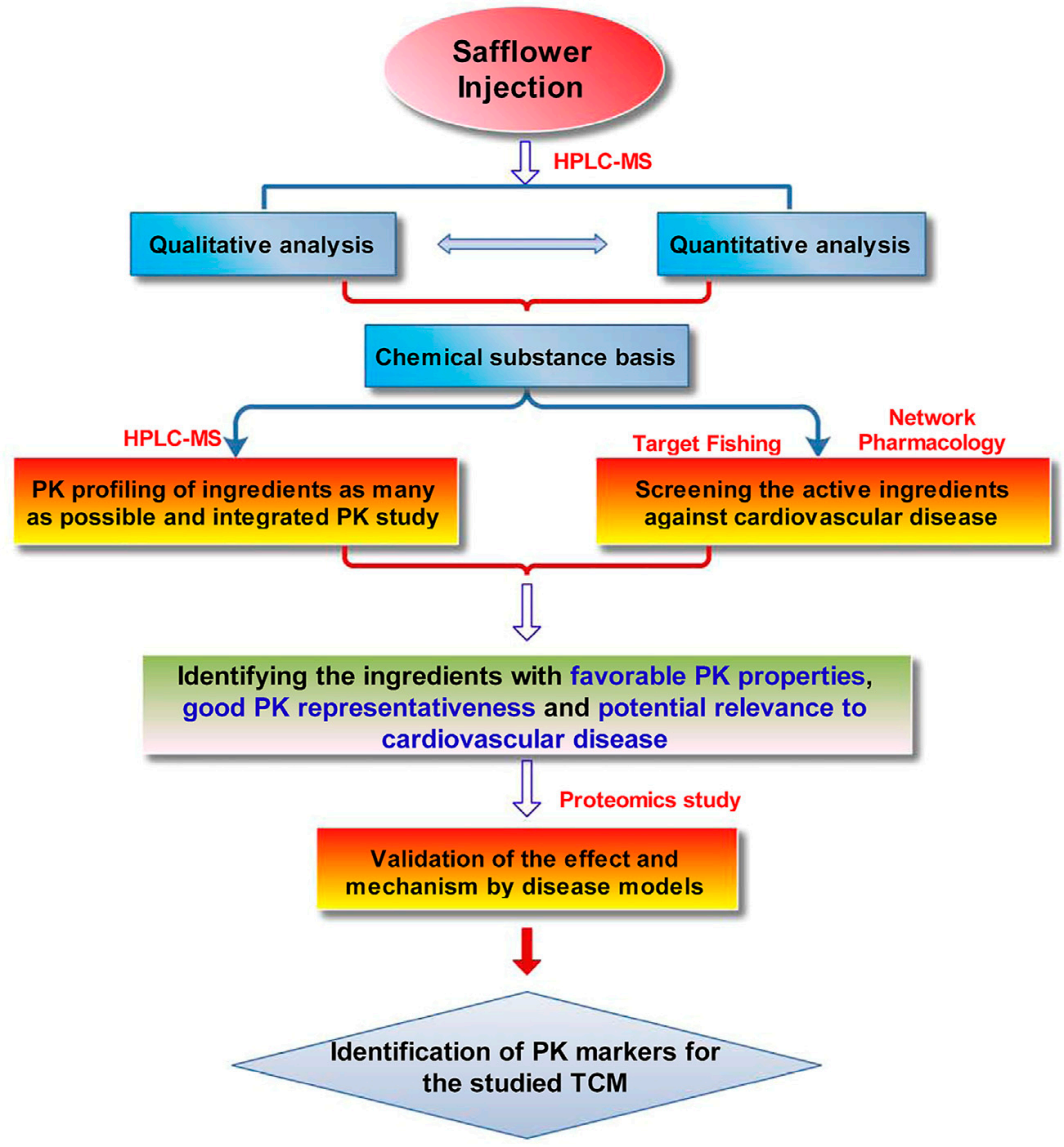
The proposed strategy for screening and identifying the PK markers of SI.

A strategy (Scheme 1) for screening and identifying PK markers of the cardio-cerebrovascular preparation of SI was put forward and verified in this work. First, qualitative and quantitative analyses were performed to reveal the chemical substance basis of the preparation *via* HPLC–MS with the aid of standard references. Second, PK profiling of multiple ingredients and integrated PK investigation for the preparation were carried out according to previous reports (Yao et al., 2018; Shi et al., 2020), to identify ingredients with favorable PK properties (easily detected at PK time points and high system exposure) and similar PK parameters to those of the multiple ingredients integrated PK in rat plasma. Third, active ingredients against CVDs in the preparation were predicted originally with target fishing and system pharmacology. Accordingly, ingredients with favorable PK properties, satisfactory PK representativeness for the preparation, and high predicted relevance to CVDs were screened as potential PK markers. Fourth, the H_2_O_2_-induced H9c2 cardiomyocyte-injured model was used to evaluate the protection of resulting ingredients against cardiomyocyte damage, followed by a proteomics study to verify the effect and mechanism of ingredients further. Finally, ingredients with satisfactory PK properties and representativeness, high relevance to myocardial damage issues, and validated therapeutic effects are selected as PK markers. The results showed that HSYA is a suitable PK marker for SI. The results of this study may provide a feasible reference for identifying PK markers of cardiovascular TCMs.

## 2 Materials and methods

### 2.1 Chemicals and reagents

SYR, HSYA, *p*-CA, SCU, *p*-hydroxybenzaldehyde (*p*-HBA), rutin (RU), quercetin, trans-cinnamic acid, kaempferol, adenosine, uridine, guanosine, cytidine, and riboflavin [internal standard (IS)] with a purity of >98% were purchased from Shanghai Ronghe Medicine Technology Development Co., Ltd. SIs (batch Nos. 19050511, 18113011, and 18101511) were manufactured by Langzhi Group Wanrong Pharmaceutical (Wanrong, Shanxi Province, China). Methanol and acetonitrile were HPLC grade (Sigma, United States). Ultra-pure water was purified by the Mini D system (Kertone, Changsha, China). Acetic acid was purchased from Aladdin (Shanghai, China). Fetal bovine serum (FBS) and trypsin were obtained from Gibco (NY, United States). Penicillin (P), streptomycin (S), and Dulbecco’s modified Eagle’s medium (DMEM, high glucose) were obtained from HyClone (United States). 3-(4,5-Dimethylthiazol-2-yl)-2,5-diphenyltetrazolium bromide (MTT) and Dimethylsulfoxide (DMSO) were provided by Aladin (Shanghai, China). 30% H_2_O_2_ solution was obtained from Sinopharm Chemical Reagent Co., Ltd., (Shanghai, China). Vitamin C (Vc) (purity: 98%) was obtained from Sigma-Aldrich Co., (St. Louis, MO, United States).

### 2.2 Experimental animals

The animal experiments were approved by the Animal Care and Use Committee of the College of Animal Science (College of Bee Science), Fujian Agriculture and Forestry University (Approval Number: PZCASFAFU21012). 250 ± 20 g of male Sprague-Dawley (SD) rats were provided by Lab Animal Center in Fujian Medical University (Fuzhou, China). The rats were acclimated to 22°C ± 2°C, relative humidity at 40%–70%, and a 12 h light and 12 h dark cycle, and the animal could access to feed and water *ad libitum*. Before administration, all rats were fasted for 12 h, but free access to water.

### 2.3 Qualitative analysis for safflower injection

HPLC analysis was carried out on an Agilent 1290 Infinity LC instrument (Agilent, Waldbronn, Germany) consisting of a binary pump, a diode-array detector, an auto-sampler and a column compartment. The samples were separated on a RD-C_18_ column (4.6 mm × 50 mm, 3.5 μm) (Zhongpu Science Inc., Fuzhou, China). The mobile phase was a stepwise gradient of water (containing 0.5% acetic acid, v/v) and acetonitrile (0 min, 98: 2; 20 min, 85: 15; 30 min, 70: 30; 50–52 min, 5: 95; 52.1–60 min, 98: 2). The column temperature was 30°C, the flow rate was 0.5 ml/min and the injection volume was 5 μl. The HPLC system was connected to an Agilent 6520 Q-TOF mass spectrometer (Santa Clara, CA, United States) equipped with an electrospray ionization (ESI) interface. Mass spectra were recorded at *m/z* 100–1000, and all masses were corrected by the internal standards provided by the Agilent Technologies (Agilent Part Number: G1969-85001) with *m/z* at 112.98559 and 1033.98811 in (–)ESI mode. The data were processed with Agilent MassHunter Workstation Software version B.06.00 (Agilent Technologies).

For qualitative analysis of the components in SI, the mixture methanol solution of the 13 standard references, including SYR, HSYA, *p*-CA, SCU, *p*-HBA, RU, quercetin, trans-cinnamic acid, kaempferol, adenosine, uridine, guanosine, and cytidine (about 10 μg/ml for each ingredient), were used in the HPLC-MS identification.

### 2.4 Quantitative analysis for safflower injection

#### 2.4.1 LC-MS/MS analysis

An LC-MS system (LC-MS 8040, Shimadzu, Japan) was used for the quantitative analysis. The chromatographic column and mobile phase were the same as qualitative analysis. A gradient program was carried out as follows: 0–5 min, 94%–80% A; 5–10 min, 80%–70% A; 10–15 min, 70%–50% A; 15.1–18 min, 5% A; 18.1–25 min, 94% A. The column temperature was set at 25°C. The flow rate was 0.5 ml/min and the injection volume was 5 μl. The ESI source conditions were as follows: block heating temperature was kept at 400°C; desolvation line temperature was set at 250°C; dry gas (nitrogen) flow rate was 15 L/min; and auxiliary gas (nitrogen) flow rate was 3 L/min. Quantification was performed using multiple reaction monitoring (MRM) by monitoring the ion-pairs of *m/z* 242.00→109.00, 243.00→200.00, 266.00→134.05, 431.00→209.20, 611.00→490.95, 121.00→91.95, 163.00→119.05, 609.00→300.00, 461.00→284.95, 147.00→102.95, and 300.90→151.00 for cytidine, uridine, adenosine, SYR, HSYA, *p*-HBA, *p*-CA, RU, SCU, trans-cinnamic acid, and quercetin, respectively.

#### 2.4.2 Preparation of working solution

The mixed standard solution was prepared by accurately weighing cytidine (1.1 mg), uridine (1.1 mg), adenosine (1.0 mg), SYR (1.1 mg), HSYA (0.9 mg), *p*-HBA (1.1 mg), *p*-CA (1.2 mg), RU (1.1 mg), SCU (1.0 mg), trans-cinnamic acid (1.0 mg), and quercetin (1.0 mg), dissolving them with 1 ml ethanol in the same 1 ml volumetric flask. Then, the above solution was further diluted with methanol to obtain a series of working solutions with different concentration levels, which were stored in a refrigerator at 4°C.

#### 2.4.3 Preparation of sample solution

An appropriate amount of the injection into a 1 ml volumetric flask, fix the volume of methanol to the scale, shake well, and use 0.45 μM microporous membrane for filtration, and the filtrate was the test solution.

### 2.5 Pharmacokinetic and integrated pharmacokinetic studies for the multiple components of safflower injection

#### 2.5.1 Animal and administration

Ten rats were divided into three groups (Low dose group, *n* = 4; medium and high groups, *n* = 3, respectively). A single dose (1, 2, and 4 ml/kg) of SI (batch No. 18113011) was injected into the tail vein. 300 μl of blood was collected by cutting tail method before dosing and at the PK time points of 0.04, 0.083, 0.17, 0.25, 0.33, 0.5, 0.75 1, 1.5, 2, 3, 4, 6, 8, and 12 h after administration. For each rat, 2 ml of physiological saline was supplemented after 30 min sampling *via* intraperitoneal injection. The plasma samples were obtained by centrifugation at 3,000 rpm for 10 min and stored at −20°C until analysis.

#### 2.5.2 Plasma sample preparation

100 μl plasma, 10 μl IS solution (10 μg/ml riboflavin) and 300 μl methanol were mixed and vortexed for 3 min, following centrifugation for 11 min (13,000 rpm at 4°C). Clear supernatant (5 μl) was injected into HPLC-MS for bioanalysis.

#### 2.5.3 LC-MS conditions

An LC-MS system (LC-MS 8040, Shimadzu, Japan) was used in the study. The sample was separated on a RD-C_18_ column (4.6 mm × 50 mm, 3.5 μm, Zhongpu Develop, China). The mobile phase incudes water with 0.1% acetic acid (A) and acetonitrile (B). The gradient program was as follows: 0–7 min, 12.5%–30% B; 7–7.1 min, 30%–50% B; 7.1–13 min, 50%–95% B, and kept at 95% B from 13 to 15 min. The column temperature, flow rate and injection volume were set at 30°C, 0.5 ml/min and 5 μl, respectively. The electrospray ionization source conditions were identical with our previous research (Shi et al., 2020). Selective ion monitoring (SIM) in negative ionization mode was carried out by monitoring the [M-H]^-^ ions at *m/z* 611 for HSYA, *m/z* 163 for *p*-CA, *m/z* 461 for SCU, *m/z* 121 for *p*-HBA, *m/z* 375 for IS, and [M + HCOO]^-^ ion at *m/z* 431 for SYR, as well as MRM was also performed by monitoring the ion pair *m/z* 609-300 with collision energy at 40 V for RU.

#### 2.5.4 Method validation

The LC-MS method was validated by the analysis performance index of the six ingredients in rat plasma (See the Supplementary Material).

#### 2.5.5 Data processing

Non-compartmental model was used to calculate the PK parameters by DAS 3.0 software (Chinese Pharmacologic Society, Beijing, China). A single-tailed *t*-test was carried out in the study.

### 2.6 Systematic pharmacology analysis for the multiple ingredients of safflower injection and molecular docking

To predict the targets of the multiple ingredients of SI, the PharmMapper on-line Server (http://www.lilab-ecust.cn/pharmmapper/) was used according to previous reports (Shi et al., 2020). 300 targets were obtained from the on-line target fishing. Further, the targets with z'-score >0 were selected to take part in the following Disease Ontology Semantic and Enrichment (DOSE) analysis and molecular docking according to our previous report (Shi et al., 2020; Xie et al., 2020; Xie et al., 2021).

### 2.7 Cell culture and protection of hydroxysafflor yellow A and syringin on cardiomyocytes injured by H_2_O_2_

H9c2 cardiomyocytes obtained from Cell Bank of the Chinese Academy of Sciences (Shanghai, China) were cultured in DMEM (high glucose) supplemented with 1% P/S and 15% FBS in 5% CO_2_ at 37°C. The cells were collected with 0.25% trypsin, followed by re-seeding in 96-well multiplates with a density of 5×10^3^ cells per well. The cell experiments were grouped as follows: normal group, model group (H_2_O_2_), administration groups and positive control groups (N-Acetyl-L-cysteine, NAC). After incubation for 24 h, the model groups were injured with 250 μM H_2_O_2_ for 2 h, while before exposed to 250 μM H_2_O_2_ for 2 h cardiomyocytes in administration groups and positive control groups were pretreated with HSYA (1, 5, and 10 μM), SYR (1, 5, and 10 μM), and NAC (1 mM) for 24 h, respectively. After that, cells were incubated with 0.5 mg/ml of MTT for 4 h under standard condition, followed by addition of 150 µl DMSO into every well. The cell supernatants were finally removed and the optical density (OD) was recorded at 570 nm using a microplate reader (Thermo Fisher Scientific, United States). The protection rate of the compounds on H9c2 cardiomyocytes was calculated as (OD value_administration group (or NAC group)_-OD value_model group_)/(OD value_normal group_-OD value_model group_) ×100%.

All data were expressed as mean ± SD and analyzed using the SPSS 18.0. Statistical comparisons between groups were performed *via* one-way ANOVA. Differences were considered significant when *p* < 0.05.

### 2.8 Quantitative proteomics

For quantitative proteomics, the 195 μM HSYA and 168 μM SYR were respectively used to treat with the cardiomyocytes injured by H_2_O_2_ to obtain apparent protection. The procedures for cell culture, group and administration are similar to those in “2.7. Cell culture and protection of HSYA and SYR on cardiomyocytes injured by H_2_O_2_” with little modification. Briefly, the difference is that H9c2 cells were seeded and cultured in a culture flask with a bottom area of 25 cm^2^ with a density 1×10^5^ cells/ml. After treatment, about 3×10^7^ cells per group were collected for the next quantitative proteomics investigation, which was done by Jingjie PTM Biolabs (Hangzhou, China). All the procedures for quantitative proteomics can be found in the section of “5. Quantitative proteomics” of the Supplementary Material.

## 3 Results and discussion

### 3.1 Chemical substance basis of safflower injection

The UV chromatogram at 265 nm and total ion chromatogram in negative mode of SI are shown in Supplementary Figure S1. Thirteen ingredients, including HSYA, SYR, *p*-CA, SCU, *p*-HBA, RU, quercetin, *trans*-cinnamic acid, kaempferol, adenosine, uridine, guanosine, and cytidine, identified *via* HPLC-Q-TOF-MS with the aid of their previous reported MS information (Fan et al., 2019) and HPLC-MS-MS with the standard references, are listed in Supplementary Tables S1, S2.

**FIGURE 1:**
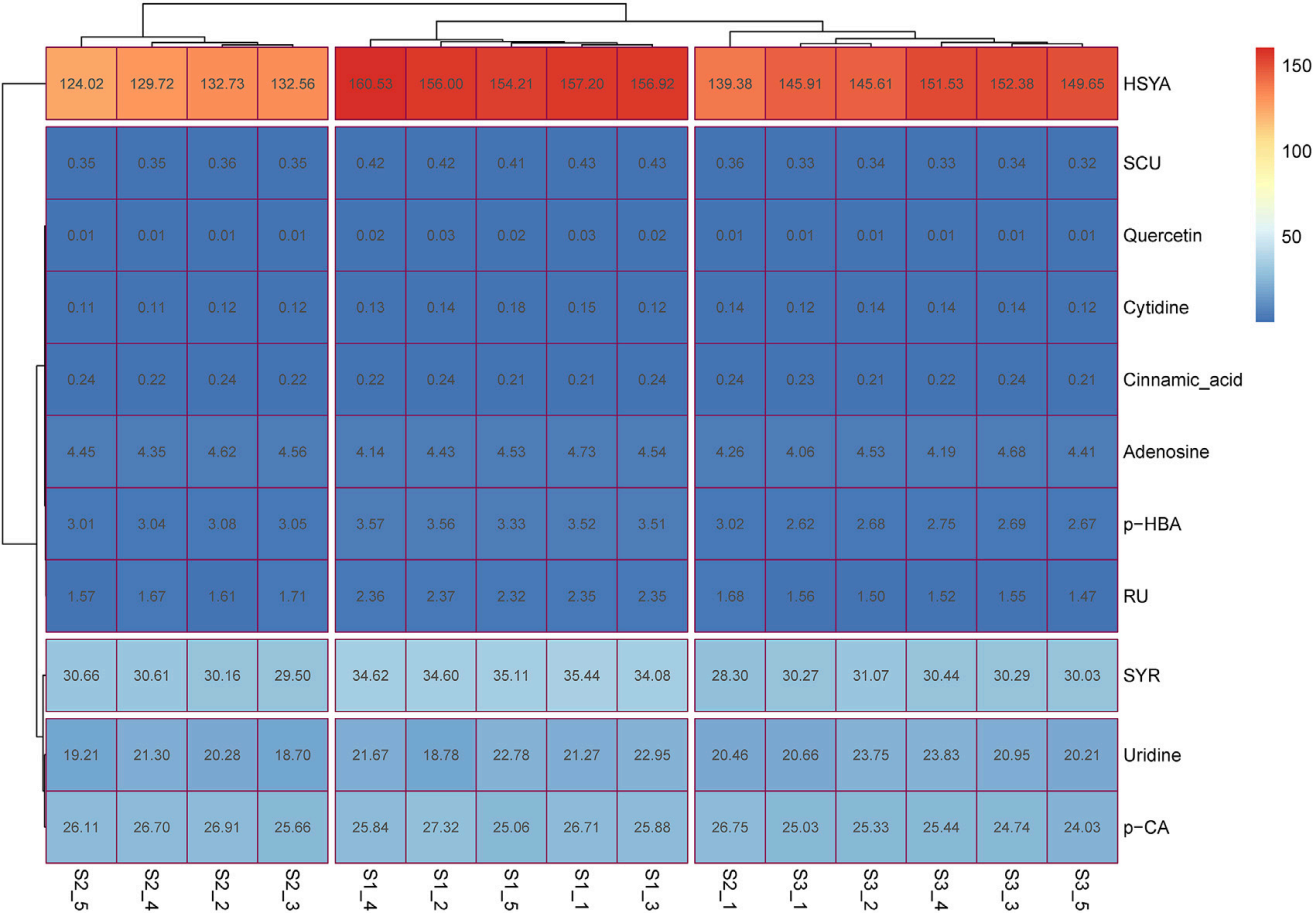
The heatmap for content determination results of multiple ingredients in SI. S1-1 to S1-5, S2-1 to S2-5, and S3-1 to S3-5 represent the five SI samples from the three batches of preparations (Nos. 19050511, 18113011, and 18101511, respectively); the number in each box represents the content of the relative ingredient in the corresponding sample with the unit of μg/ml.

Contents of guanosine and kaempferol were very low (below 0.5 ng/ml) after the preliminary experiment. The remaining 11 ingredients, including SYR, HSYA, *p*-CA, SCU, *p*-HBA, RU, quercetin, *trans*-cinnamic acid, adenosine, uridine, and cytidine, were then considered quantified. As shown in Supplementary Figure S2 and Supplementary Tables S3–S5, the developed HPLC-MS/MS method was suitable and applied to determine the content of the 11 ingredients for three batches of SI. The heatmap in Figure 1 demonstrated that HSYA presents the highest content (62% *via* normalization method), followed by the contents of SYR (11.0%), *p*-CA (9.0%), and uridine (13.5%) in all the three batches of investigated samples. The seven other ingredients can be clustered into one group because of their low contents *via* normalization method. These findings suggested that the main chemical substances of SI consist of HSYA, SYR, *p*-CA, and uridine, which account for about 95.5% of the total amount of the 11 ingredients according to the normalization method.

### 3.2 Pharmacokinetic and integrated pharmacokinetic study

Only six ingredients, including HSYA, *p*-CA, SCU, *p*-HBA, SYR, and RU, can be detected 1 h after administration (2 ml/kg of SI) in a preliminary test. Concentration levels of the seven other ingredients (quercetin, *trans*-cinnamic acid, kaempferol, adenosine, uridine, guanosine, and cytidine) were below their LODs (about 0.5 ng/ml) and cannot be determined at the PK time point of 15 min after administration. Accordingly, we focused on the PK behavior of six ingredients in SI, namely, HSYA, SYR, *p*-CA, SCU, *p*-HBA, and RU, due to the easily detectable property in rat plasma.

Methodological validation, such as selectivity, calibration curves, precision, accuracy, extraction recovery, matrix effect, and stability, was performed on the six compounds in plasma for bioanalysis. Typical SIM or MRM chromatograms for the six compounds and IS in blank, spiked, and drug plasma after administration for 5 min are presented in Supplementary Figure S3. The absence of interference indicated the acceptable selectivity of the method.

All calibration curves are summarized in Supplementary Table S6. Linearity (*r* ^2^ > 0.995) was satisfactory and within the tested range. As shown in Supplementary Table S7, RSD values were 0.23%–6.90% and 1.14%–4.70% while RE values were −5.00%–7.42% and −1.56%–5.58% for the respective intra- and interday precisions of all six analytes. This finding suggested that the proposed method is accurate and reliable. Extraction recoveries for the six analytes were in the range of 84.12%–103.45%, with RSD values of 2.62%–10.99%, and matrix effects were in the range of 99.77%–121.41%, with RSD values of 1.09%–12.20% (Supplementary Table S8). These results demonstrated that the proposed method is reliable for bioanalysis. The results of prepreparation, postpreparation, freezing, thawing, and long-term stability experiments are listed in Supplementary Table S9. These values indicated that analytes are stable and can be detected with acceptable accuracy (RE within ±15%).

**FIGURE 2:**
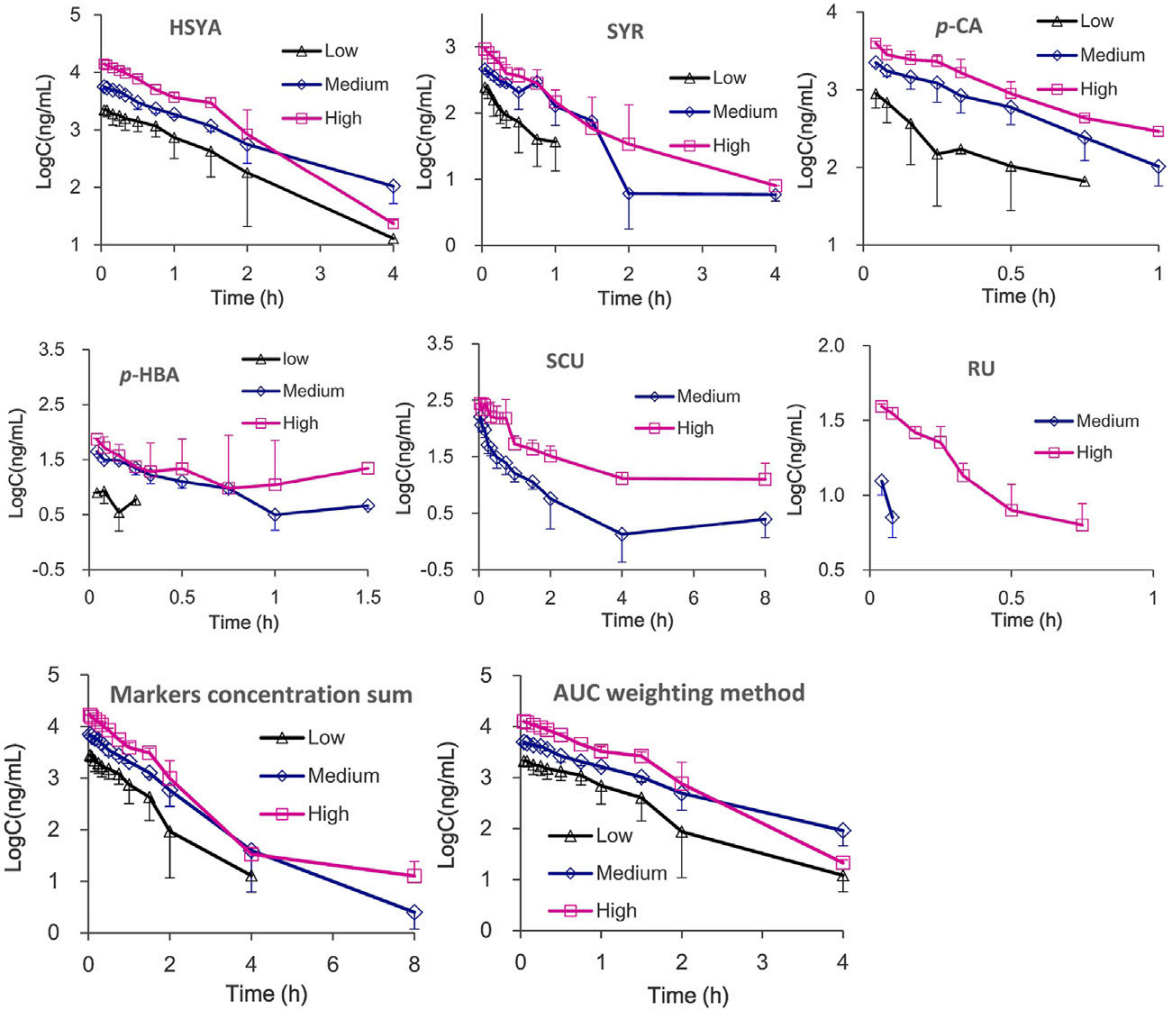
The mean plasma drug concentration–time curves and integrated plasma drug concentration-time curves of six ingredients after *i.v.,* administration of SI with high (4 ml/kg, Mean ± SD, *n* = 3), medium (2 ml/kg, Mean ± SD, *n* = 3), and low (1 ml/kg, Mean ± SD, *n* = 4) dosages.

The plasma drug concentration–time curves for the six ingredients (*i.v.*, 1, 2, and 4 ml/kg of SI) are shown in Figure 2. The calculated PK parameters (Table 1) showed that HSYA, SYR, *p*-CA, *p*-HBA, and RU are eliminated rapidly (0.08 h ≤ *t*_1/2_ ≤ 0.64 h), while SCU was eliminated slowly with a *t*_1/2_ ≥ 2.58 h in rats. Meanwhile, the order of AUC values is HSYA > SYR > *p*-CA > SCU > *p*-HBA > RU. Notably, the systematic exposure extent of HSYA was 6-10 times higher than that of SYR (See the AUC values in Table 1). Meanwhile, SCU, *p*-HBA, and RU can’t be detected behind 15 min after *i.v*., low dose administration (1 ml/kg of SI is equivalent to a mean dosage of normal adult).

**TABLE 1:** The PK parameters of six ingredients in rat plasma after *i.v.*, administration of SI with low and high dosages (Mean ± SD).

Analyte	Group	*t*_1/2_	C_max_	AUC_0-t_	AUC_0-∞_	Vd	Cl	MRT_0-t_	MRT_0-∞_
		(h)	(ng/ml)	(h·ng/ml)	(h·ng/ml)	(L/kg)	[L/(h·kg)]	(h)	(h)
SYR	Low	0.25 ± 0.09	259.50 ± 59.35	100.67 ± 57.37	115.88 ± 70.51	0.21 ± 0.07	0.69 ± 0.43	0.27 ± 0.10	0.38 ± 0.16
	Medium	0.61 ± 0.25	465.33 ± 42.92	363.24 ± 81.55	367.67 ± 78.73	0.31 ± 0.20	0.34 ± 0.07	0.69 ± 0.07	0.75 ± 0.14
	High	0.53 ± 0.23	925.33 ± 38.55	585.69 ± 94.42	591.71 ± 96.56	0.31 ± 0.11	0.41 ± 0.07	0.67 ± 0.21	0.71 ± 0.23
HSYA	Low	0.47 ± 0.07	2,624.50 ± 660.17	2,479.11 ± 1,312.76	2,490.30 ± 1,317.76	0.09 ± 0.04	0.13 ± 0.07	0.76 ± 0.19	0.78 ± 0.19
	Medium	0.64 ± 0.08	5,628.33 ± 405.14	5,074.18 ± 328.93	5,461.77 ± 202.42	0.09 ± 0.01	0.10 ± 0.00	0.79 ± 0.14	0.97 ± 0.06
	High	0.46 ± 0.12	14,077.33 ± 17.21	11,015.37 ± 1,025.71	11,453.34 ± 668.38	0.06 ± 0.02	0.09 ± 0.01	0.70 ± 0.09	0.78 ± 0.11
*p*-CA	Low	0.08 ± 0.05	305.00 ± 147.31	62.45 ± 50.04	67.71 ± 57.20	0.11 ± 0.03	1.21 ± 0.76	0.10 ± 0.05	0.12 ± 0.07
	Medium	0.22 ± 0.06	715.00 ± 85.75	265.09 ± 89.73	277.26 ± 84.05	0.13 ± 0.07	0.40 ± 0.13	0.27 ± 0.03	0.33 ± 0.01
	High	0.21 ± 0.05	1,255.00 ± 69.07	432.92 ± 44.53	469.10 ± 38.10	0.14 ± 0.03	0.45 ± 0.04	0.25 ± 0.04	0.32 ± 0.04
SCU	Low	—	—	—	—	—	—	—	—
	Medium	2.58 ± 1.84	162.20 ± 17.41	89.76 ± 21.01	104.73 ± 31.46	0.05 ± 0.02	0.01 ± 0.01	1.46 ± 0.64	2.90 ± 2.42
	High	2.84 ± 1.19	327.33 ± 60.05	305.74 ± 12.47	358.05 ± 33.97	0.03 ± 0.01	0.01 ± 0.00	1.57 ± 0.68	2.96 ± 1.49
*p*-HBA	Low	—	—	—	—	—	—	—	—
	Medium	0.48 ± 0.11	44.67 ± 5.13	20.15 ± 4.40	23.41 ± 5.21	0.35 ± 0.04	0.53 ± 0.11	0.43 ± 0.04	0.67 ± 0.11
	High	0.39 ± 0.35	74.33 ± 2.52	35.19 ± 31.15	44.04 ± 45.17	0.34 ± 0.09	0.99 ± 0.66	0.32 ± 0.25	0.53 ± 0.53
RU	Low	—	—	—	—	—	—	—	—
	Medium	—	—	—	—	—	—	—	—
	High	0.24 ± 0.04	39.33 ± 1.53	12.38 ± 1.37	15.10 ± 2.23	0.30 ± 0.02	0.86 ± 0.13	0.22 ± 0.03	0.36 ± 0.06
Con.sum	Low	0.47 ± 0.07	3,167.75 ± 816.03	2,652.33 ± 1,429.09	2,663.49 ± 1,433.99	0.12 ± 0.06	0.18 ± 0.10	0.73 ± 0.18	0.75 ± 0.18
	Medium	0.67 ± 0.03	7,028.20 ± 327.77	6,118.67 ± 358.43	6,121.59 ± 360.32	0.12 ± 0.01	0.13 ± 0.01	0.92 ± 0.06	0.93 ± 0.06
	High	0.61 ± 0.19	16,654.00 ± 83.47	12,888.28 ± 601.42	12,905.39 ± 600.72	0.10 ± 0.04	0.12 ± 0.01	0.76 ± 0.05	0.77 ± 0.05
AUC integrated	Low	0.47 ± 0.07	2,479.02 ± 623.45	2,331.72 ± 1,235.38	2,342.22 ± 1,240.07	0.09 ± 0.04	0.13 ± 0.07	0.76 ± 0.19	0.78 ± 0.19
	Medium	0.64 ± 0.08	4,977.67 ± 350.18	4,314.31 ± 162.32	4,651.87 ± 328.87	0.09 ± 0.01	0.10 ± 0.01	0.79 ± 0.15	0.98 ± 0.05
	High	0.45 ± 0.13	12,613.33 ± 15.31	9,842.06 ± 914.28	10,231.64 ± 594.87	0.06 ± 0.02	0.09 ± 0.01	0.69 ± 0.09	0.78 ± 0.11
Effect weighting integrated	Low	0.47 ± 0.07	2,565.28 ± 644.69	2,419.83 ± 1,281.52	2,430.74 ± 1,286.40	0.09 ± 0.04	0.13 ± 0.07	0.76 ± 0.19	0.78 ± 0.19
	Medium	0.80 ± 0.18	5,498.98 ± 395.38	5,178.98 ± 544.93	5,337.30 ± 637.82	0.11 ± 0.02	0.10 ± 0.01	0.91 ± 0.11	1.03 ± 0.13
	High	0.58 ± 0.20	13,737.03 ± 17.63	11,273.38 ± 1,221.77	11,861.16 ± 262.38	0.07 ± 0.02	0.09 ± 0.00	0.70 ± 0.09	0.83 ± 0.17

Low dose group, *n* = 4; medium and high groups, *n* = 3, respectively. The plasma drug concentration was low, so the PK, parameters could not be calculated.

Meanwhile, the multiple integrated PK of HSYA, SYR, *p*-CA, SCU, *p*-HBA, and RU was used with integrated methods (Yao et al., 2017; Shi et al., 2018; Yao et al., 2018; Shi et al., 2020), namely, “plasma drug concentration sum” and “AUC weighting integrated” methods. As shown in Figure 2 and Table 1, differences between the individual HSYA PK and the integrated PK obtained from the two integrated methods were insignificant for nearly all the PK parameters. The results suggested that PK characteristics of HSYA can reflect the integrated *in vivo* process of multiple ingredients of SI because its PK parameters are similar to those of the multiple-ingredient integrated PK in rat plasma.

### 3.3 System pharmacology

**FIGURE 3:**
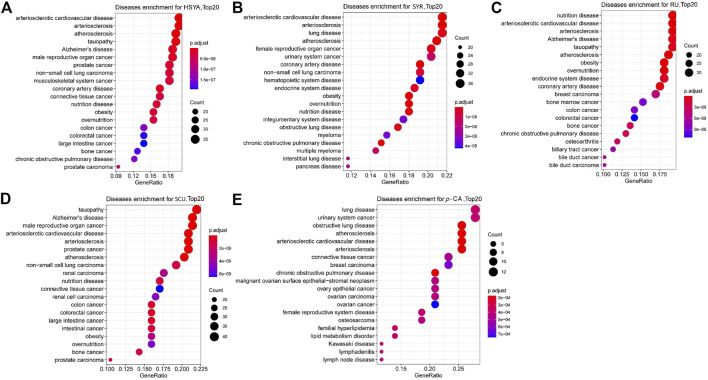
DOSE analysis plots for the five ingredients, **(A)** HSYA, **(B)** SYR, **(C)** RU, **(D)** SCU and **(E)** *p*-CA with favorable PK properties. The top 20 diseases relative to the corresponding ingredient and potential targets are shown in the graphs.

System pharmacology is an excellent tool for determining the effects and mechanisms of multiple-component systems, such as TCM (Cheng et al., 2022). PK studies showed that ingredients, such as HSYA, *p*-CA, SCU, *p*-HBA, and SYR, (except for RU) demonstrate favorable PK properties (easily detected at PK time points and high system exposure). These six ingredients were utilized in the systematic pharmacology study. Potential targets with a z'-score > 0 for ingredients HSYA, *p*-CA, SCU, *p*-HBA, SYR, and RU were obtained through target fishing. These ingredients were subjected to DOSE analysis using R-package. The remaining five ingredients, except for *p*-HBA, can collect a series of diseases using their relative potential targets. As shown in Figure 3, HSYA and SYR can show “rank 1” relevance to arteriosclerotic cardiovascular disease, while *p*-CA, SCU, and RU can be highly correlated to lung disease, tauopathy, and nutrition disease. Meanwhile, *p*-CA, SCU, and RU can also be correlated with arteriosclerotic cardiovascular disease. However, their contents in SI and exposure extents in systematic circulation are minimal. In addition, a DOSE graph for *p*-HBA is unavailable due to the low relevance of its potential target proteins to diseases. The results suggested that HSYA and SYR may be the main active components that exert the effects of anti-CVDs for SI.

**FIGURE 4:**
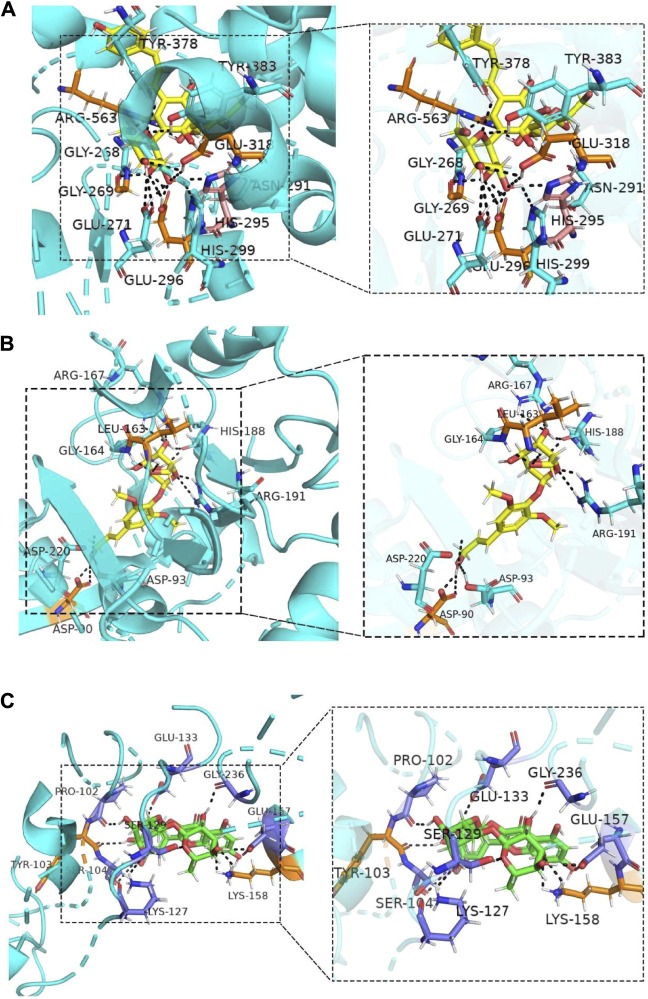
Molecular docking combinations of **(A)** HSYA, **(B)** SYR and **(C)** RU with their receptor targets, LTA-4H, SDH, and CTSB.

Molecular docking was performed on the five ingredients of HSYA, SYR, *p*-CA, SCU, and RU using their potential target proteins, which are correlated with arteriosclerotic cardiovascular disease in the DOSE analysis, to support this conjecture on active components of SI against CVDs further. As shown in Figure 4 and Supplementary Table S10, “rank 1” affinity targets of HSYA, SYR, *p*-CA, SCU, and RU (docking total score >7.0 and binding free energy ΔG ≤ −4 kcal/mol) are leukotriene A4 hydrolase (LTA4H), sorbitol dehydrogenase (SDH), deoxycytidine kinase, cell division protein kinase 2, and cathepsin B (CTSB), respectively, while *p*-HBA fails to find a target protein with a docking total score of >7.0 and binding free energy of ΔG ≤ −4 kcal/mol. Particularly, LTA4H and SDH are key targets for cardiovascular disease treatment (Sandanayaka et al., 2010; Tang et al., 2010). Therefore, the molecular docking results further supported the conjecture of roles of HSYA and SYR on anti-CVDs. CTSB can be used as an indicator for myocardial ischemia/reperfusion (I/R) injury (Qiao et al., 2021), thereby suggesting the possible positive effect of RU on CVDs. The consistency between molecular docking and DOSE analysis results confirmed that HSYA and SYR may be the main active components that exert effects of anti-CVDs in SI.

### 3.4 Protective effect of hydroxysafflor yellow A and syringin on cardiomyocytes injured by H_2_O_2_

**FIGURE 5:**
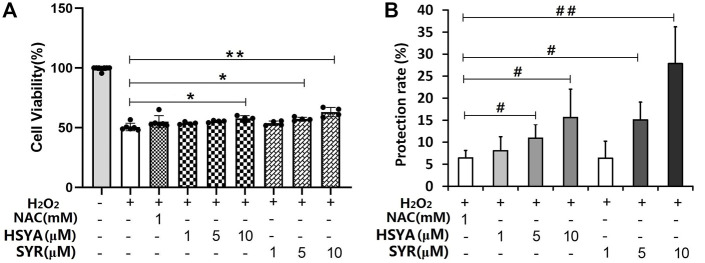
**(A)** Cell viability and **(B)** protection rate for the treatment of HSYA and SYR on cardiomyocytes injured by H_2_O_2_. ^*^ *p* < 0.05 and ^**^ *p* < 0.01 vs. model group (mean ± SD, *n* = 4); ^#^ *p* < 0.05 and ^##^ *p* < 0.01 vs. NAC group (mean ± SD, *n* = 3).

The protection of HSYA, SYR, *p*-CA, SCU, *p*-HBA, and RU was evaluated on cardiomyocytes injured by H_2_O_2_ to provide the direct evidence on the anti-CVD activity of ingredients. The PK test revealed that the detected mean maximum plasma concentrations for HSYA, SYR, *p*-CA, SCU, *p*-HBA, and RU are 14.07 (23 μM), 0.93 (2.5 μM), 1.25 (8.5 μM), 0.327 (0.7 μM), 0.074 (0.61 μM), and 0.039 (0.06 μM) μg/mL, respectively, after the administration of 4 ml/kg of SI, at which dosage the plasma concentration Cmax value of HSYA in rats is very close to that in human beings when administrating a single dose of 140 mg of HSYA injection to an adult (Yang et al., 2009). These ingredients with their maximum detected plasma concentration levels in protection tests were evaluated preliminarily. The results presented that 23 μM of HSYA and 2.5 μM of SYR achieve protection rates of about 26% and 10%, respectively, while the positive drug (1 mM of NAC) shows a protection rate of 6.45% to injured cardiomyocytes. However, 8.5 μM of *p*-CA, 0.7 μM of SCU, 0.61 μM of *p*-HBA, and 0.06 μM of RU demonstrated the absence of protection. Furthermore, HSYA and SYR were compared at the three concentration levels of 1, 5, and 10 μM on the basis of cardiomyocytes injured by H_2_O_2_. As shown in Figure 5A, 5- and 10-μM SYR and 10-μM HSYA groups show higher cell viability than the model group (*p* < 0.05 or *p* < 0.01). Although no statistical difference exists between the same concentration of HSYA and SYR on protection rates, both compounds show a concentration-dependent anti-H_2_O_2_ injury effect on cardiomyocytes (Figure 5B). The results directly confirmed that HSYA and SYR can be the main active substances exerting cardiomyocyte protection, especially when the normal dosage of SI is administered to human beings.

### 3.5 Quantitative proteomics

**FIGURE 6:**
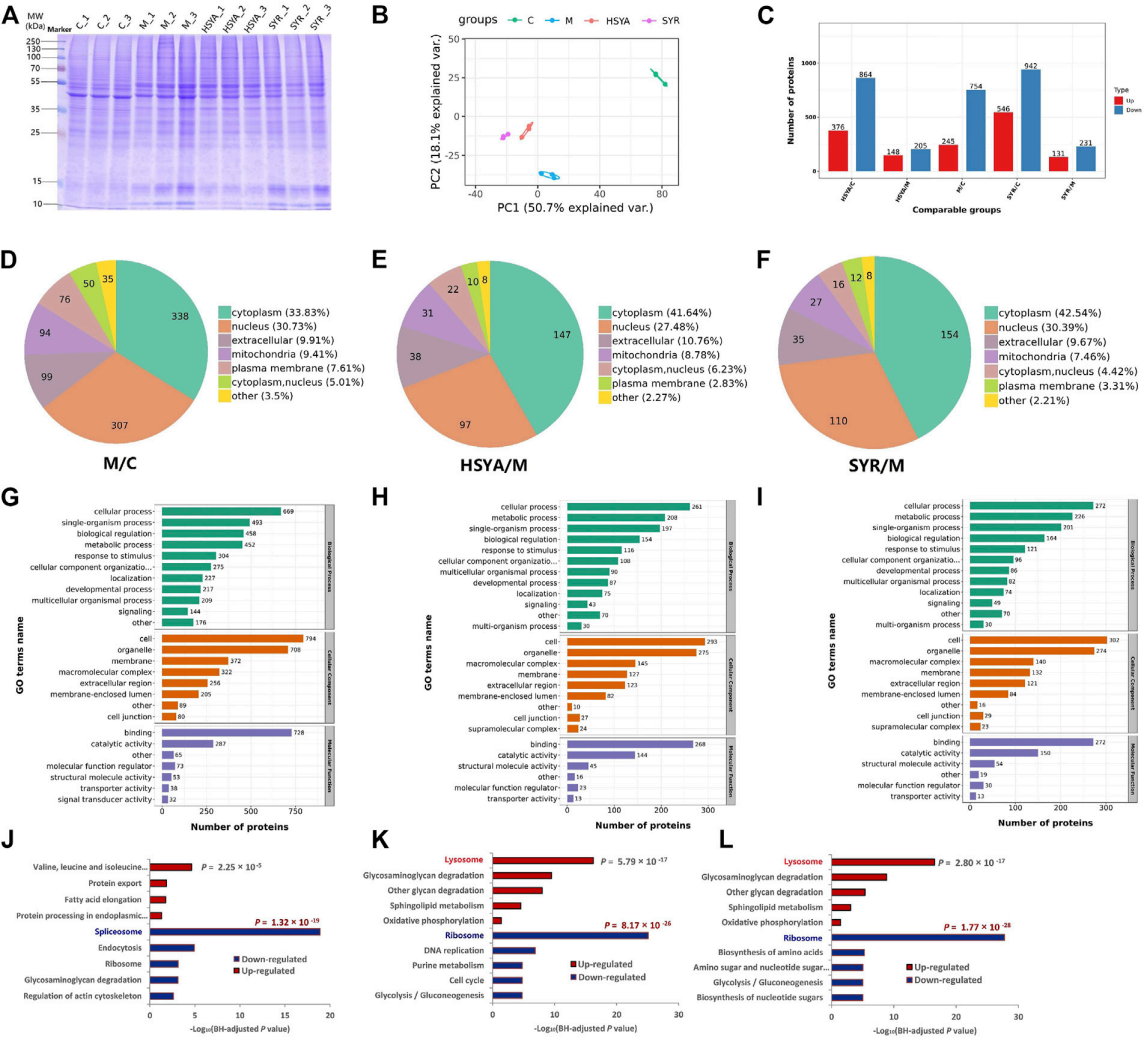
**(A)** Represents the SDS-PAGE analysis for the total proteins of C, M, HSYA and SYR group. **(B,C)** show the PCA analysis score plot and the difference proteins numbers, respectively for M vs. C, HSYA vs. M and SYR vs. M groups. **(D–F)** show the difference expression proteins location in the comparisons for M vs. C, HSYA vs. M and SYR vs. M groups, respectively. **(G–I)** show the GO annotation results of the difference expression proteins for M vs. C, HSYA vs. M and SYR vs. M groups, respectively. **(J–L)** show the downregulation and upregulation pathways for M vs. C, HSYA vs. M and SYR vs. M groups, respectively.

Twelve samples, including three control (C), three model (M), three HSYA-treated (HSYA), and three SYR-treated (SYR) groups, were monitored *via* SDS-PAGE analysis before the TMT quantitative proteomics study. As shown in Figure 6A, protein bands were clear and uniform, the parallelism of each lane in the group was satisfactory, and the electrophoretic behavior between groups was different when observed with the naked eye. This finding preliminary confirmed that significant differences exist in the protein expression between various treatment groups as well as provided support to the succeeding quantitative proteomics study.

The quantitative proteomics study was carried out according to our previous report (Li et al., 2022). Relative quantitative values of proteins in two samples were compared *via t*-test. The protein expression was significantly upregulated when the *p*-value and fold change of the differential expression were <0.05 and ≥1.5, respectively. The protein expression was significantly downregulated when the *p*-value and the fold change of the differential expression were <0.05 and ≤1/1.5, respectively. A total of 7,380 proteins were identified and 6,595 proteins were quantified in the twelve samples for the four groups. The identified difference proteins between groups were subjected further to principal component analysis (PCA). The results showed that the cluster degree of samples in each group is high and samples are significantly separated between different treatment groups (Figure 6B). Notably, both HSYA and SYR groups showed recovery in the direction of PC2 toward the C group, thereby suggesting their possible interference effect against H_2_O_2_ injury to cardiomyocytes. As shown in Figure 6C and Supplementary Figure S4, 245 proteins were upregulated (such as Atpefg, Ndufa10, and Atp6l) and 754 proteins were downregulated (such as Tpm3, Parv, and Msn) in the M group compared with those in the C group; 148 proteins significantly upregulated (such as Glg1, Esl1, and Tpm3) and 205 proteins significantly downregulated (such as Grb2, Mapkapk2, and Cdh3) in the HSYA group compared with those in the M group, while 131 proteins (such as Rrs1, Ndufa8, and Lmnb2) upregulated and 231 proteins (such as Mcm4, Map2k1, and Dab2ip) downregulated in the SYR group compared with those in the M group. The subcellular structure location was analyzed using these proteins as objectives. As shown in Figures 6D–F, all treatments, including H_2_O_2_ injury, H_2_O_2_ injury plus HSYA interference, and H_2_O_2_ injury plus SYR interference, mainly changed the protein expression located at the cytoplasm by 33.83% in the M group vs. C group, 41.64% in the HSYA group vs. M group, and 42.54% in the SYR group vs. M group as well as the nucleus by 30.73%, 27.48%, and 30.39%, respectively.

Furthermore, Gene Ontology (GO) classification was performed to understand the functional classification of all differentially expressed proteins (DEPs) between groups. In the annotation of biological process (BP), the majority of DEPs involved in the cellular process under all comparisons of M versus C (Figure 6G), HSYA versus M (Figure 6H), and SYR versus M (Figure 6I) groups. The majority of DEPs in the annotation of cellular component (CC) came from cells and organelles under all comparisons. Molecular function (MF) analysis demonstrated that the majority of DEPs are concerned with binding and catalytic activities. The GO analysis indicated that DEPs play predominant roles in the structural composition of ribosome, structural molecular activity, and peptide biosynthetic process for the comparisons between HSYA and M groups as well as SYR and M groups (Supplementary Figure S5). Notably, the results of GO enrichment for both of HSYA versus M and SYR versus M are very similar with consideration for BP, CC, and MF enrichments. This finding suggested that the interference effect of HSYA and SYR is also similar to the metabolism pathways of injured cardiomyocytes *via* H_2_O_2_.

Finally, Kyoto Encyclopedia of Genes and Genomes (KEGG) enrichment of DEPs was carried out between groups. As shown in Figure 6J, H_2_O_2_ treatment mainly downregulated the activity of the spliceosome pathway and upregulated the valine, leucine, and isoleucine degradation pathways due to the stress alternation of cardiomyocytes against injury. However, the interference of both HSYA and SYR mainly downregulated the ribosome pathway and upregulated the lysosome pathway in H_2_O_2_-injured cardiomyocytes (Figures 6K,L). The significant downregulation of the ribosome pathway in long-lived individuals (LLIs) (Xiao et al., 2022) suggested that downregulating the ribosome pathway can delay cell aging and death. The upregulation of the lysosome function can promote autophagy of damaged cells and then play a role in myocardial protection (Gu et al., 2020). These results demonstrated that both HSYA and SYR can protect cardiomyocytes from death and the effect mechanism can be primarily related to the downregulation of the ribosome pathway and the upregulation of the lysosome pathway by the two ingredients. In addition, two pathways related to energy metabolism, including glycolysis/gluconeogenesis and oxidative phosphorylation, were downregulated and upregulated, respectively, and contributed to the effect and partly accounted for the mechanism of both HSYA and SYR against cardiomyocyte injury.

Quantitative proteomics further confirmed the cardiomyocyte protection of the two ingredients and revealed their effect mechanisms related to multiple pathways, including ribosome, lysosome, glycolysis/gluconeogenesis, and oxidative phosphorylation.

### 3.6 Identification of pharmacokinetic markers and integrated pharmacokinetic with markers for safflower injection

**FIGURE 7:**
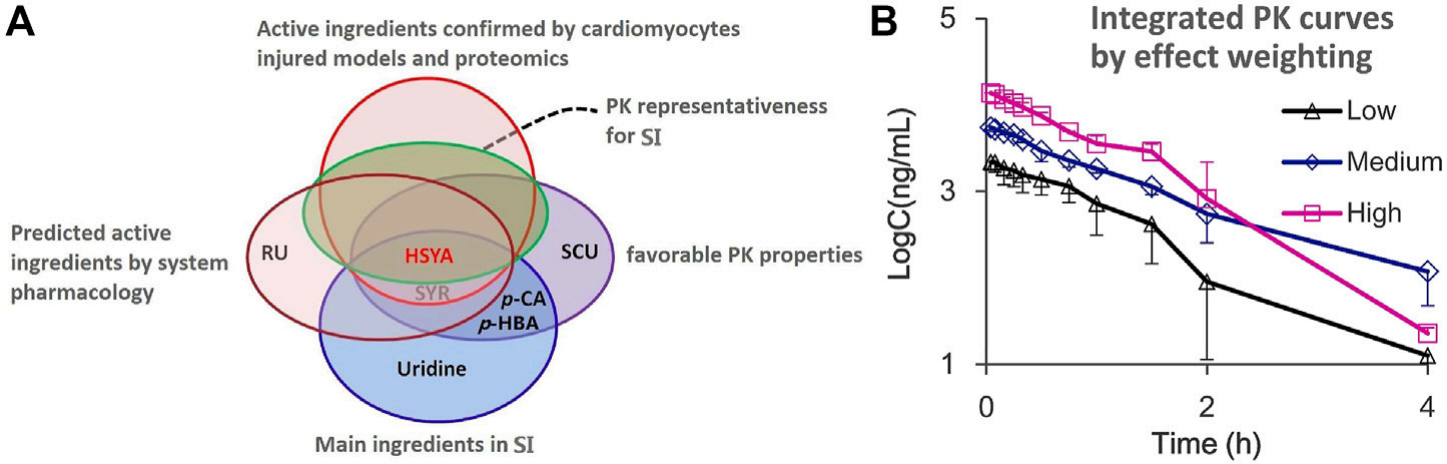
**(A)** Venn diagram of PK marker identification for SI and **(B)** the integrated drug plasma concentration-time curves by the effect weighting integrated method with the plasma concentration of two ingredients HSYA and SYR after i.v., administration of SI with high (4 ml/kg, Mean ± SD, *n* = 3), medium (2 ml/kg, Mean ± SD, *n* = 3), and low (1 ml/kg, Mean ± SD, *n* = 4) dosages.

As shown in Figure 7A, chemical substance basis analysis indicated that the SI mainly consists of HSYA, SYR, *p*-CA, and uridine, and the component with absolute predominance in content is HSYA. The multiple-component PK study further confirmed that five ingredients, including HSYA, SYR, *p*-CA, SCU, and *p*-HBA, present favorable PK properties while only HSYA shows very similar PK parameters (no significant difference) to multiple ingredient-integrated PK. This finding is consistent with the PK representative potential of the whole SI preparation *in vivo*. System pharmacology predicted that HSYA and SYR may be the main anti-CVD active ingredients, which are validated by the experiments of protective effect study on cardiomyocytes injured by H_2_O_2_. Ultimately, the proteomics study further confirmed the anti-CVD effect and mechanisms of the two ingredients HSYA and SYR. According to the results, HSYA can be considered a PK marker to represent the whole *in vivo* process of multiple ingredients of SI not only for its favorable PK properties but also satisfactory cardiomyocyte protection when 4 ml/kg of SI is administered. This dosage is clinically equivalent to that of pure HSYA injection for adults. Although the systematic exposure of SYR is over 10 times lower than that of HSYA when administered, its plasma drug concentration within the first 15 min after administration is about 1–1.25 μM, which achieves cardiomyocyte protection rates of 6%–10%. Therefore, SYR should be considered an important ingredient due to its possible contribution to the therapeutic effect of SI and noted in the whole characterization preparation PK behavior. On this basis, the effect weighting-integrated PK was explored with our previous suggestion (Shi et al., 2018). Briefly, the area under the protection rate-drug concentration curve (*AUC*_*effect*_) was applied to calculate the coefficient of effect weighting and the integrated plasma drug concentrations as follows:

$$\omega i=\frac{AUCi_{effect}}{\overset{n}{\underset{1}{\sum }}AUC_{effect}},$$ (1)

$$\overset{n}{\underset{1}{\sum }}AUC_{effect}=AUC1_{effect}+AUC2_{effect}+\ldots +AUCi_{effect}+\ldots +AUCn_{effect},$$ (2)

$$C_{Z}=\omega_{1}C_{1}+\omega_{2}C_{2}+\omega_{3}C_{3}+\ldots +\omega_{n}C_{n},$$ (3)

where *ω* is the effect weighting coefficient, *i* is a certain ingredient, *n* is the number of investigated ingredients, *C*_*1*_–*C*_*n*_ are the concentrations of each ingredient at one PK time point, and *C*_*Z*_ is the integrated plasma drug concentration. At present, only the two ingredients (HSYA and SYR) are involved in the integrated PK study due to their positive anticardiomyocyte injury effect and favorable PK properties. The PK profiles indicated that the plasma drug concentration range of HSYA and SYR is 0.02–23 and 0.02–2.5 μM and the calculated average *AUC*_*effect*_ within their plasma drug concentration ranges are 473.19% and 12.18% µM, respectively. According to Eqs 1, 2, the calculated effect weighting coefficients of HSYA and SYR are 97.5 and 2.5, respectively. As results, with the PK profiles for HSYA and SYR (Figure 2) and above Eq. 3, the calculated integrated concentrations, PK curves, and parameters are presented in Figure 7B and Table 1.

Notably, although the resulting effect weighting coefficient for HSYA is 39 times higher than that for SYR under the condition of adult administration dosage, the therapeutic role of SYR should not be neglected due to its good protection on cardiomyocytes injured in the preparation of SI. Table 1 presents that differences in PK parameters between HSYA and the effect weighting integrated curves are insignificant. This finding supports the PK representative role of HSYA for the overall *in vivo* process characterization of SI, and of course, HSYA and SYR can be recommended as quality markers (Lu et al., 2022) for quality control of safflower preparations.

## 4 Conclusion

A novel strategy for screening PK markers for the cardiovascular herbal medicine SI was put forward and practiced in this work by combining system pharmacology, multi-ingredient PK, and quantitative proteomics study. The ingredient HSYA with favorable PK properties, satisfactory PK representativeness for the preparation, and high predicted relevance to CVDs was considered the optimal candidate and then screened as a potential PK marker. The H_2_O_2_-induced H9c2 cardiomyocyte-injured model was used to evaluate the protection of ingredients against myocardial damage. The results confirmed that HSYA and SYR are the main active ingredients in SI. The subsequent proteomics study further validated the effect and mechanism of ingredients against myocardial damage. Finally, the ingredient HSYA with satisfactory PK properties and representativeness, high relevance to myocardial damage issues, and verified pharmacological effects were identified as the PK marker for the preparation while considering the effect weighting under the condition of adult administration dosage. The results of this study may provide a reference for the characterization of PK markers for other cardiovascular TCMs.

## Data Availability

The data presented in the study are deposited in the ProteomeXchange Consortium *via* the iProX partner repository (https://www.iprox.cn/page/home.html), accession number PXD038145 (http://proteomecentral.proteomexchange.org/cgi/GetDataset?ID=PXD038145).
