# Supplementary material for: Identification of pharmacokinetic markers for safflower injection using a combination of system pharmacology, multicomponent pharmacokinetics, and quantitative proteomics study
Source: Front Pharmacol. 2022 Nov 23;13:1062026. doi: 10.3389/fphar.2022.1062026 (PMC9727182; doi:10.3389/fphar.2022.1062026)
Supplement: Supplementary file 1 [file DataSheet1.docx]

**Supplementary Material for**

Identification of pharmacokinetic markers for safflower injection using a combination of system pharmacology, multicomponent pharmacokinetics, and quantitative proteomics study

**1. Quantitative analysis for SI**

| **Peak no.** | **Retention**  **Time (min)** | **Formula** | **Calculated mass (*m/z*)** | **TOF–MS [M−H]^−^**/  **[M+CH_3_COO]^−^** | **△ppm** | **Assigned identity** |
| --- | --- | --- | --- | --- | --- | --- |
| **1** | 1.25 | C_10_H_13_N_5_O_5_ | 283.0917 | [M−H]^−^ 282.0836 | 2.64 | Guanosine |
| **2** | 1.25 | C_9_H_13_N_3_O_5_ | 243.1815 | [M−H]^−^ 242.1744 | 7.92 | Cytidine |
| **3** | 1.42 | C_10_H_13_N_5_O_4_ | 267.1107 | [M−H]^−^ 266.1028 | -2.50 | Adenosine |
| **4** | 2.71 | C_9_H_12_N_2_O_6_ | 244.0688 | [M−H]^−^ 243.0615 | 2.92 | Uridine |
| **5** | 14.87 | C_17_H_24_O_9_ | 371.1329 | [M+CH_3_COO]^−^  431.1543 | 0.75 | Syringin (SYR) |
| **6** | 15.75 | C_27_H_32_O_16_ | 612.1694 | [M−H]^−^ 611.1606 | -0.55 | HSYA |
| **7** | 14.80 | C_7_H_6_O_2_ | 122.0359 | [M−H]^−^ 121.0285 | 7.52 | *p*-Hydroxybenzaldehyde (*p*-HBA) |
| **8** | 20.82 | C_9_H_8_O_3_ | 164.0464 | [M−H]^−^ 163.0390 | 5.04 | *p*-Coumaric acid (*p*-CA) |
| **9** | 22.70 | C_27_H_30_O_16_ | 610.3187 | [M−H]^−^ 609.3030 | 0.04 | Rutin (RU) |
| **10** | 23.19 | C_21_H_18_O_12_ | 462.1567 | [M−H]^−^ 461.1487 | 5.88 | Scutellarin (SCU) |
| **11** | 34.66 | C_15_H_10_O_7_ | 302.1105 | [M−H]^−^ 301.1198 | 3.10 | Quercetin |
| **12** | 37.95 | C_9_H_8_O_2_ | 148.0369 | [M−H]^−^ 147.0292 | 4.71 | trans-Cinnamic acid |
| **13** | 38.28 | C_15_H_10_O_6_ | 286.2128 | [M−H]^−^ 285.2055 | -3.61 | Kaempferol |

TABLE S1 Identification results of ingredients in SI by HPLC-Q-TOF-MS

**
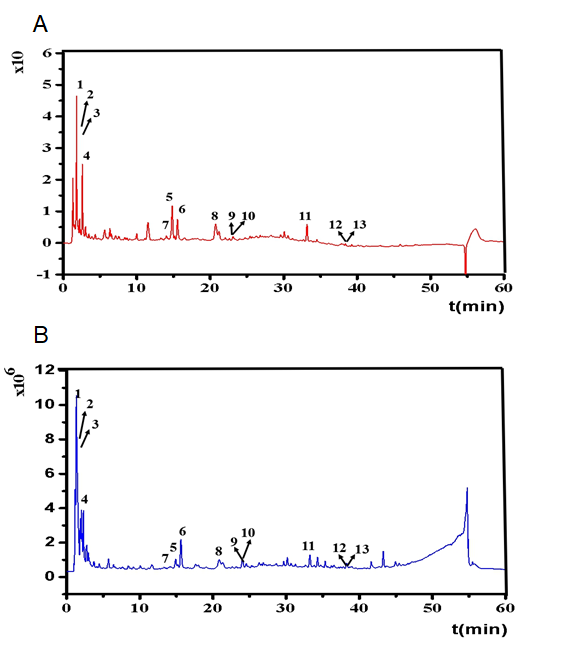
**

**FIGURE S1** HPLC-UV chromatogram at 265 nm (A) and total ion chromatogram in negative mode of SI (B).

| **Peak no.** | **Formula** | **(-) MS/MS fragments ions** | **Assigned identity** |
| --- | --- | --- | --- |
| **1** | C_10_H_13_N_5_O_5_ | 282 [M−H]^−^; MS^2^: 151.00 (100) | Guanosine |
| **2** | C_9_H_13_N_3_O_5_ | 242 [M−H]^−^; MS^2^: 109.00 (100) | Cytidine |
| **3** | C_10_H_13_N_5_O_4_ | 266 [M−H]^−^; MS^2^: 134.05 (100) | Adenosine |
| **4** | C_9_H_12_N_2_O_6_ | 243 [M−H]^−^; MS^2^: 200.00 (100) | Uridine |
| **5** | C_17_H_24_O_9_ | 431 [M+CH_3_COO]^−^; MS^2^: 209.20 (100) | SYR |
| **6** | C_27_H_32_O_16_ | 611 [M−H]^−^; MS^2^: 490.95 (100) | HSYA |
| **7** | C_7_H_6_O_2_ | 121 [M−H]^−^; MS^2^: 91.95 (100) | *p*-HBA |
| **8** | C_9_H_8_O_3_ | 163 [M−H]^−^; MS^2^: 119.05 (100) | *p*-CA |
| **9** | C_27_H_30_O_16_ | 609 [M−H]^−^; MS^2^: 300.00 (100) | RU |
| **10** | C_21_H_18_O_12_ | 461 [M−H]^−^; MS^2^: 284.95 (100) | SCU |
| **11** | C_15_H_10_O_7_ | 301 [M−H]^−^; MS^2^: 151.00 (100) | Quercetin |
| **12** | C_9_H_8_O_2_ | 147 [M−H]^−^; MS^2^: 102.95 (100) | trans-Cinnamic acid |
| **13** | C_15_H_10_O_6_ | 285 [M−H]^−^; MS^2^: 93.00 (100) | Kaempferol |

TABLE S2 Identification results of ingredients in SI by HPLC-MS/MS

**2. Quantitative analysis for SI**

*2.1. Specificity experiment*

Compare the MRM spectra of blank solvent, blank solvent plus standard and injection to investigate the specificity. As shown in Figure S2, there is no interference peak observed in the chromatograms of blank solvent, standard solution and injection sample.


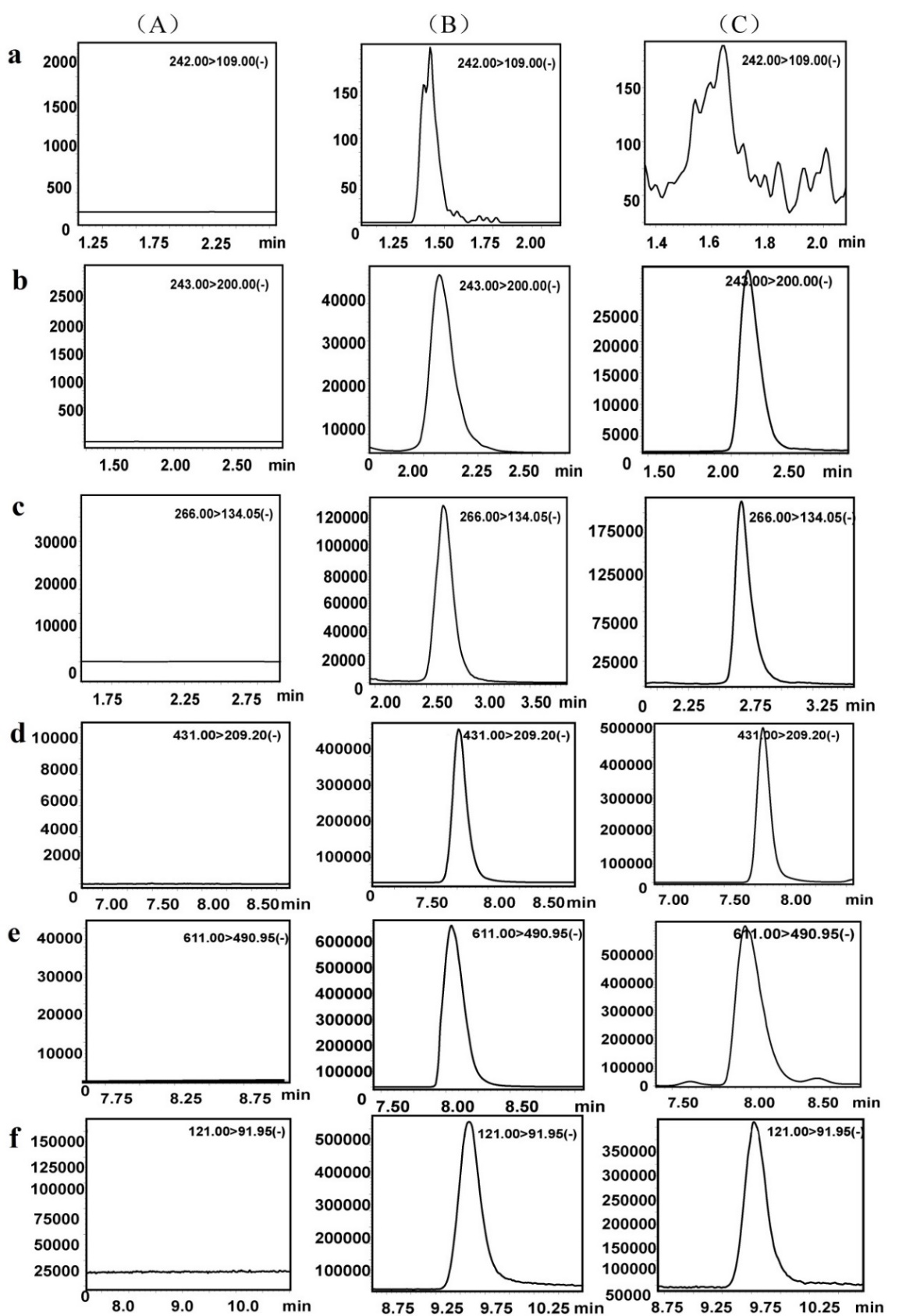


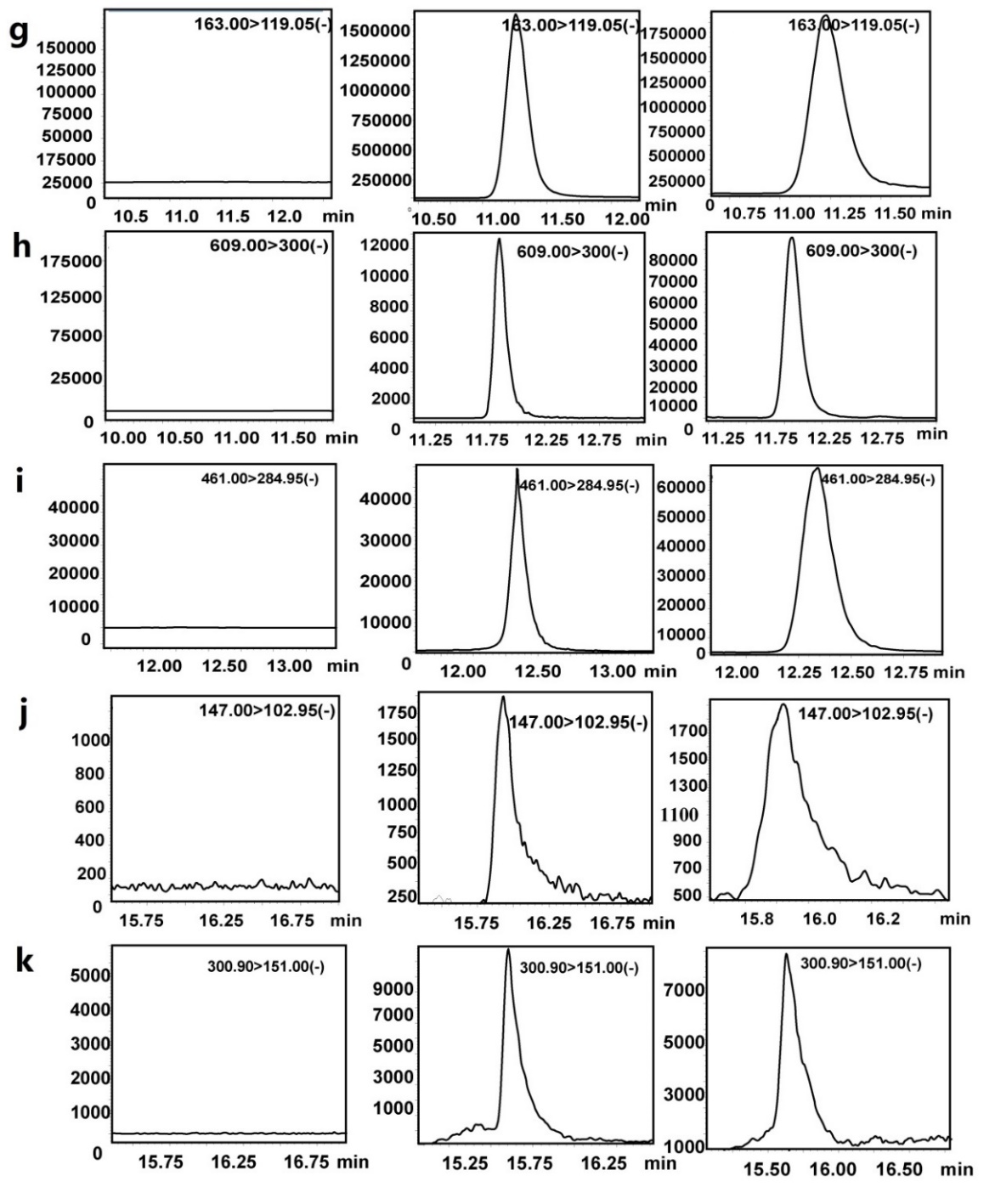


**FIGURE S2** The typical MRM chromatograms of (A) blank solvents, (B) the mixed standard solution and (C) SI. a. Cytidine, b. Uridine, c. Adenosine, d. SYR, e. HSYA, f. *p*-HBA, g. *p*-CA, h. RU, i. SCU, j. trans-cinnamic acid, and k. quercetin.

*2.2. Linearity, LOD and LOQ*

Series concentration of the working solution was analyzed by LC-MS/MS analysis to investigate the linear range, correlation coefficient (r^2^), detection limit (LOD) and quantitative limit (LOQ) of 11 compounds. The calibration curves were generated by plotting the corresponding peak area of each ingredient versus the concentration of the relative standard (*C*) by the linear regression. The LOD and LOQ were defined as the concentration with the signal/noise ≥ 3 and ≥ 10, respectively. As shown in Table S3, All the 11 studied ingredients have good linear relationships between their concentrations and peak areas within the studied concentration ranges (r^2^>0.999).

**Table S3** Calibration curves, LODs and LOQs for determination of the eleven ingredients

| **Analyte** | **Regression equation**  **(μg/mL)** | **Linear range**  **(μg/mL)** | **r^2^** | **LOD**  **(ng/mL)** | **LOQ**  **(ng/mL)** | **Repeatability**  **RSD (%)** | **Stability**  **RSD (%)** |
| --- | --- | --- | --- | --- | --- | --- | --- |
| Cytidine | Y=71773.8X+857.00 | 0.0020-15.6 | 0.9994 | 1.0 | 2.0 | 1.48 | 1.85 |
| Uridine | Y=11357.8X+578.67 | 0.0310-62.5 | 0.9995 | 15.3 | 31.0 | 3.54 | 2.81 |
| Adenosine | Y=27014.0X+48.37 | 0.0038-62.5 | 0.9993 | 1.0 | 3.8 | 2.86 | 2.74 |
| SYR | Y=130449.0X+76.91 | 0.0020-62.5 | 0.9992 | 0.5 | 2.0 | 1.37 | 2.76 |
| HSYA | Y=35633.9X-15.21 | 7.8150-250 | 0.9995 | 1.0 | 15.3 | 1.02 | 2.56 |
| *p*-HBA | Y=762157.0X+121069 | 0.0310-62.5 | 0.9993 | 15.3 | 31.0 | 2.46 | 4.11 |
| *p*-CA | Y=636203X+111031 | 0.245-62.5 | 0.9998 | 3.8 | 15.3 | 2.24 | 3.29 |
| RU | Y=329375X+4965.79 | 0.245-31.25 | 0.9994 | 0.3 | 1.0 | 2.89 | 2.31 |
| SCU | Y=1248000X+655329 | 0.015-15.6 | 0.9997 | 0.5 | 1.0 | 2.10 | 4.42 |
| trans-Cinnamic acid | Y=26551.2X+13539.9 | 0.245-62.5 | 0.9996 | 0.5 | 1.0 | 3.68 | 1.86 |
| Quercetin | Y=2694710X+24016.7 | 0.0095-0.24 | 0.9997 | 0.5 | 1.0 | 3.20 | 2.51 |

*2.3. Precision, repeatability, stability and recovery*

The intra-day and inter-day precision were tested by assaying the mixed authentic standard solution samples within 1 day and 3 days in five times, respectively. The relative standard deviation (RSD) was taken as a measure of precision. The results were in the range of 0.95%-4.15% and 0.59%-4.53% for the intra-day and inter-day precision, respectively (Table S4).

**Table S4** Precisions of 11 compounds in SI

| **Analyte** | **Precisions** | | | | | | |
| --- | --- | --- | --- | --- | --- | --- | --- |
|  | **Intra-day (n=5)** | | | **Inter-day (n=5)** | | | |
|  | **Mean±SD (μg/ml)** | **RSD (%)** | | | **Mean±SD (μg/ml)** | | **RSD (%)** |
| Cytidine  Uridine  Adenosine | 0.0201±0.0084 | | 4.15 | | 0.0198±0.0011 | 4.53 | |
|  | 14.8039±0.3778 | | 2.55 | | 14.9288±0.2183 | 1.46 | |
|  | 15.0544±0.1437 | | 0.95 | | 15.0782±0.1282 | 0.85 | |
| SYR  HSYA  *p*-HBA | 15.1581±0.2870 | | 1.89 | | 15.0316±0.1638 | 1.09 | |
|  | 20.2525±0.2252 | | 1.11 | | 20.3556±0.2993 | 1.44 | |
|  | 20.4821±0.2515 | | 1.23 | | 20.5794±0.1979 | 0.96 | |
| *p*-CA  RU  SCU | 15.0122±0.2358 | | 1.57 | | 14.9842±0.3053 | 2.04 | |
|  | 15.0484±0.1807 | | 1.20 | | 15.1876±0.0900 | 0.59 | |
|  | 0.3082±0.0094 | | 3.04 | | 0.3046±0.0101 | 3.32 | |
| trans-Cinnamic acid | 14.9554±0.1911 | | 1.28 | | 14.8156±0.1952 | 1.32 | |
|  |  | |  | |  |  | |
| Quercetin | 0.0203±0.0008 | | 4.02 | | 0.0204±0.0005 | 2.68 | |

To evaluate the repeatability of the developed assay, five samples from the same batch of SI were treated according to the sample preparation procedure and analyzed with the established method. The RSD of each compound ranged from 1.02% to 3.68% (Table S3).

The stability was confirmed with a sample of diluted SI at room temperature and analyzed at 0, 2, 4, 8, 12, 24, and 48 h. The RSD values were all less than 5% (Table S3).

The recovery was determined by spiking accurately known amounts of the authentic standard solution to the diluted SI samples, and then the mixture solutions were analyzed with the described method. The content of each component was calibrated from the corresponding calibration curve. The recovery was in the range of 92.4 – 104.3%, with RSD no more than 3.59% (Table S5).

**Table S5** Recoveries of 11 compounds in SI (n=5)

| **Analyte** | **Recoveries (n=5)** | | | | |
| --- | --- | --- | --- | --- | --- |
|  | **Original** | **Added** | **Detected** | **Recovery** | **RSD** |
|  | **(μg/mL)** | **(μg/mL)** | **(μg/mL)** | **(%)** | **(%)** |
| Cytidine | 4.822 | 4.777 | 9.551 | 99.5 | 2.63 |
| Uridine | 21.546 | 21.684 | 42.387 | 98.1 | 2.80 |
| Adenosine | 0.142 | 0.147 | 0.299 | 103.5 | 1.63 |
| SYR | 31.177 | 31.007 | 60.898 | 97.9 | 0.57 |
| HSYA | 149.685 | 149.167 | 305.558 | 102.2 | 1.68 |
| *p*-HBA | 3.154 | 3.172 | 6.599 | 104.3 | 3.14 |
| *p*-CA | 24.127 | 24.026 | 44.516 | 92.4 | 2.12 |
| RU | 1.843 | 1.856 | 3.719 | 100.5 | 1.34 |
| SCU | 0.422 | 0.444 | 0.857 | 98.9 | 3.59 |
| trans-Cinnamic acid | 0.258 | 0.251 | 0.492 | 96.7 | 2.16 |
|  |  |  |  |  |  |
| Quercetin | 0.010 | 0.010 | 0.021 | 103.5 | 3.43 |

**3. Methodological validation results for six ingredients of SI in rat plasma**

The LC-MS method was validated by linearity, limits of detection (LOD), limits of quantification (LOQ), precision, accuracy, extraction recovery, matrix effect and stability of the 6 ingredients in rat plasma. Calibration curves were plotted using weighted linear regression of the peak area ratio of analyte to the IS against the corresponding nominal concentration of the analyte. LOD and LOQ were defined as the detectable concentration at which the ratio of signal to noise was more than 3 and 10, respectively. Intra- and inter-day accuracy and precision were assessed by detecting quality control (QC) samples using five replicates of rat samples at three concentration levels on one or three validation days, respectively. Accuracy and precision were expressed by relative error (RE) and relative standard deviation (RSD), respectively. The extraction recovery and matrix effect of the 6 components at above-mentioned three concentration levels were determined. Stability was assessed under the condition of placing un-treated plasma samples at room temperature for 6 h, and extracted plasma samples in auto-sampler for 10 h, as well as placing plasma samples within freeze-thaw three cycles and at −80 °C for 15 days.


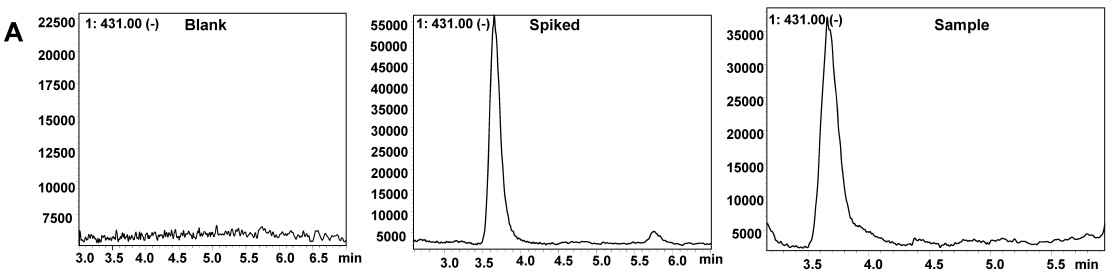


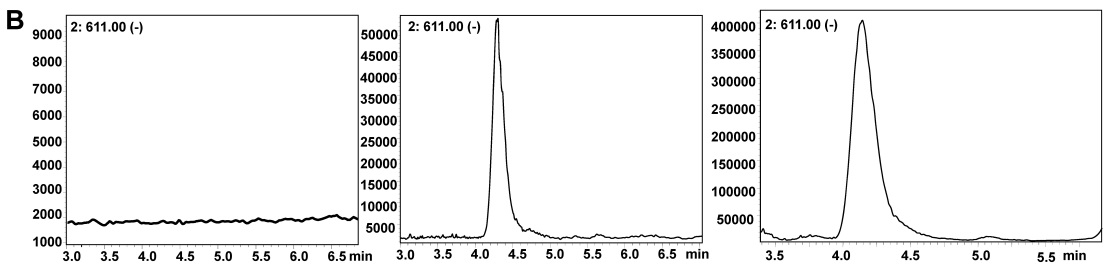


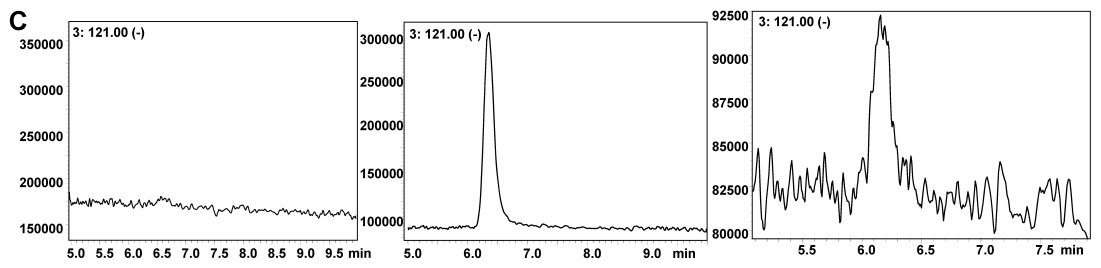


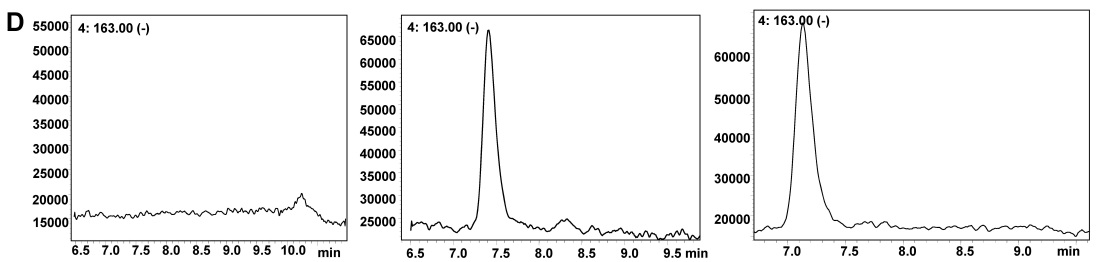


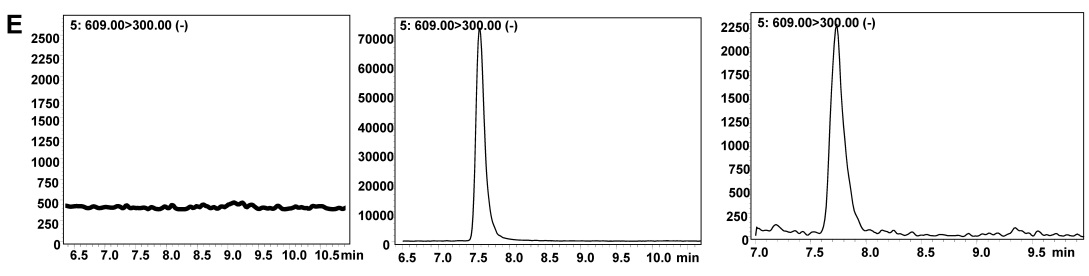


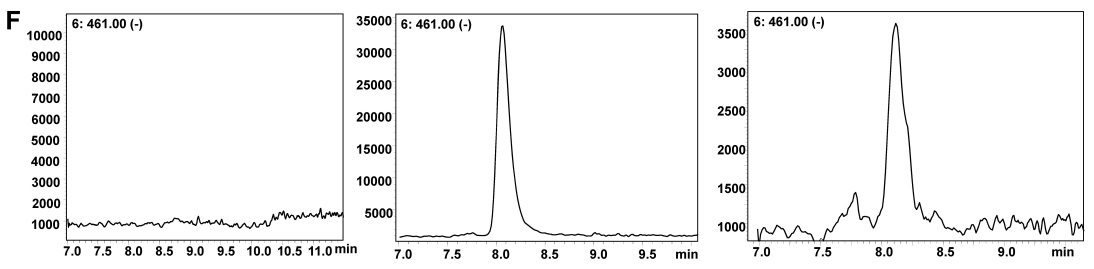


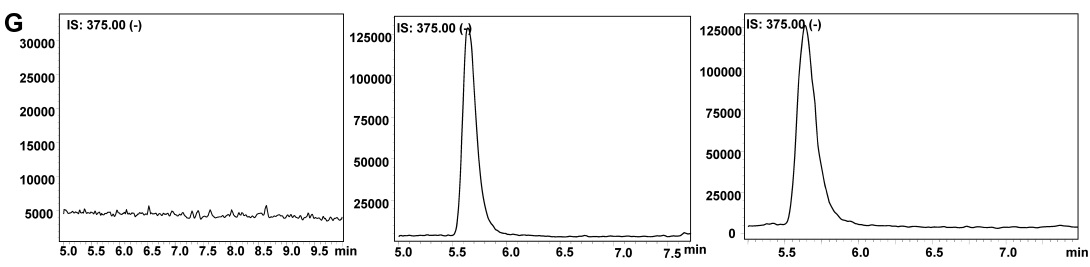


**FIGURE S3** The SIM or MRM chromatograms of 6 compounds and IS in negative mode. A, SYR; B, HSYA; C, *p*-HBA; D, *p*-CA; E, RU; F, SCU and G, IS.

**Table S6** The regression equation, linear range, r^2^, LOD and LOQ of 6 compounds in rat plasma

| **Compound** | **Regression equation** | **Linear range（μg/mL）** | **r^2^** | **LOD**  **(ng/mL)** | **LOQ**  **(ng/mL)** |
| --- | --- | --- | --- | --- | --- |
| SYR | Y = 1.886X + 0.030 | 0.012-12.5 | 0.996 | 6.0 | 12.0 |
| HSYA | Y = 1.578X + 0.013 | 0.012-12.5 | 0.996 | 6.0 | 12.0 |
| *p*-HBA | Y = 5.579X + 0.095 | 0.012-12.5 | 0.996 | 6.0 | 12.0 |
| *p*-CA | Y = 1.211X + 0.087 | 0.048-12.5 | 0.997 | 24.0 | 48.0 |
| RU | Y = 2.544X + 0.016 | 0.012-12.5 | 0.996 | 6.0 | 12.0 |
| SCU | Y = 1.432X + 0.010 | 0.012-12.5 | 0.997 | 6.0 | 12.0 |

**Table S7** The precision and accuracy of 6 compounds of SI in rat plasma

| **Analyte** | **Concentration (ng/mL)** | **Intra-day (n=5)** | | | **Inter-day (n=5)** | | |
| --- | --- | --- | --- | --- | --- | --- | --- |
|  |  |  |  |  |  |  |  |
|  |  | **Detected** | **RSD** | **Accuracy** | **Detected** | **RSD** | **Accuracy** |
|  |  | **Mean±SD (ng/mL)** | **(%)** | **(RE, %)** | **Mean±SD**  **(ng/mL)** | **(%)** | **(RE, %)** |
| SYR | 40 | 38.00±1.58 | 4.16 | -5.00 | 39.88±1.86 | 4.66 | -0.31 |
|  | 400 | 399.20±6.69 | 1.67 | -0.20 | 399.21±4.54 | 1.14 | -0.20 |
|  | 10000 | 9939.60±46.00 | 0.46 | -0.60 | 10058.00±159.84 | 1.59 | 0.58 |
| HSYA | 40 | 38.80±1.79 | 4.61 | -3.00 | 39.38±1.54 | 3.92 | -1.56 |
|  | 400 | 400.60±5.13 | 1.28 | 1.50 | 399.71±5.33 | 1.33 | -0.07 |
|  | 10000 | 10100.20±61.33 | 0.61 | 1.02 | 10203.75±190.25 | 1.86 | 2.04 |
| *p*-HBA | 40 | 41.00±2.00 | 4.88 | 2.50 | 40.63±1.59 | 3.91 | 1.56 |
|  | 400 | 399.60±5.55 | 1.39 | -0.10 | 403.50±5.64 | 1.40 | 0.88 |
|  | 10000 | 10064.00±34.20 | 0.34 | 0.64 | 10315.25±359.21 | 3.48 | 3.21 |
| *p*-CA | 150 | 151.40±2.97 | 1.96 | 0.93 | 150.00±2.73 | 1.82 | 0.00 |
|  | 400 | 409.60±5.32 | 1.30 | 2.40 | 403.64±6.91 | 1.71 | 0.91 |
|  | 10000 | 10562.60±208.33 | 1.97 | 5.63 | 10490.42±260.39 | 2.48 | 4.90 |
| RU | 40 | 41.00±2.83 | 6.90 | 2.50 | 40.38±1.71 | 4.23 | 0.94 |
|  | 1000 | 1039.40±19.01 | 1.83 | 3.94 | 1027.29±34.14 | 3.32 | 2.73 |
|  | 10000 | 10195.00±23.18 | 0.23 | 1.95 | 10229.25±213.97 | 2.09 | 2.29 |
| SCU | 40 | 40.40±2.07 | 5.13 | 1.00 | 39.56±1.86 | 4.70 | -1.10 |
|  | 1000 | 1060.20±28.54 | 2.69 | 6.02 | 1051.71±42.06 | 4.00 | 5.17 |
|  | 10000 | 10741.80±44.74 | 0.42 | 7.42 | 10551.83±233.21 | 2.21 | 5.58 |

**Table S8** The extraction recoveries and matrix effect of 6 compounds of SI in rat plasma

| **Analyte** | **Concentration (ng/mL)** | **Extraction recovery (n=5)** | | **Matrix effect (n=5)** | |
| --- | --- | --- | --- | --- | --- |
|  |  |  |  |  |  |
|  |  | **Mean± SD (%)** | **RSD (%)** | **Mean±SD (%)** | **RSD (%)** |
|  |  |  |  |  |  |
| SYR | 40 | 84.12±7.58 | 8.70 | 103.35±10.83 | 10.48 |
|  | 400 | 97.05±8.85 | 9.91 | 102.97±5.20 | 5.05 |
|  | 10000 | 97.39±3.48 | 3.58 | 99.77±1.09 | 1.09 |
| HSYA | 40 | 99.35±10.86 | 10.93 | 104.84±12.80 | 12.20 |
|  | 400 | 95.04±10.44 | 10.99 | 112.25±10.38 | 9.25 |
|  | 10000 | 100.93±3.49 | 3.46 | 103.01±7.80 | 7.57 |
| *p*-HBA | 40 | 103.16±3.54 | 3.43 | 100.88±7.99 | 7.92 |
|  | 400 | 93.53±7.39 | 7.90 | 113.09±10.85 | 9.59 |
|  | 10000 | 95.27±2.57 | 2.70 | 111.21±9.03 | 8.12 |
| *p*-CA | 150 | 99.36±7.21 | 7.26 | 103.83±2.68 | 2.58 |
|  | 400 | 103.45±10.56 | 10.20 | 107.33±10.12 | 9.43 |
|  | 10000 | 102.25±2.68 | 2.62 | 112.85±3.93 | 3.48 |
| RU | 40 | 95.72±4.02 | 4.20 | 101.99±3.26 | 3.19 |
|  | 1000 | 100.07±3.67 | 3.67 | 105.36±4.98 | 4.73 |
|  | 10000 | 95.25±8.71 | 9.15 | 112.38±10.72 | 9.54 |
| SCU | 40 | 97.17±4.01 | 4.13 | 104.35±3.69 | 3.54 |
|  | 1000 | 93.53±6.67 | 7.13 | 115.38±8.49 | 7.36 |
|  | 10000 | 88.82±3.16 | 3.56 | 121.41±7.87 | 6.49 |

**Table S9** The stability of 6 compounds of SI in rat plasma

| **Analyte** | **Spiked (ng/mL)** | **6h at room  temperature** | | **10h in  auto-sampler  vials** | | **Freeze-thaw  stability (three cycles)** | | **Long-term  stability (-80**℃**,15 days)** | |
| --- | --- | --- | --- | --- | --- | --- | --- | --- | --- |
|  |  |  |  |  |  |  |  |  |  |
|  |  |  |  |  |  |  |  |  |  |
|  |  | **RE** | **RSD** | **RE** | **RSD** | **RE** | **RSD** | **RE** | **RSD** |
|  |  | **(%)** | **(%)** | **(%)** | **(%)** | **(%)** | **(%)** | **(%)** | **(%)** |
| SYR | 40 | -2.50 | 7.48 | -12.50 | 10.69 | 1.50 | 1.35 | -9.50 | 10.77 |
|  | 400 | 3.65 | 0.91 | 1.61 | 1.27 | 2.65 | 2.02 | 0.30 | 1.15 |
|  | 10000 | 1.55 | 0.78 | 1.41 | 3.24 | 2.30 | 2.59 | 0.98 | 6.64 |
| HSYA | 40 | -10.50 | 6.37 | -13.75 | 11.23 | 2.50 | 4.56 | 3.00 | 7.56 |
|  | 400 | 4.80 | 1.00 | 1.00 | 2.18 | 3.30 | 0.79 | 0.80 | 1.07 |
|  | 10000 | -4.43 | 3.33 | 0.06 | 3.32 | 4.92 | 1.09 | -3.85 | 4.44 |
| *p*-HBA | 40 | -10.50 | 4.14 | -2.50 | 5.13 | 1.00 | 2.21 | 14.50 | 3.91 |
|  | 400 | -3.70 | 1.34 | 0.63 | 2.13 | 4.05 | 0.64 | 0.45 | 1.44 |
|  | 10000 | -3.91 | 2.53 | 4.23 | 1.78 | -3.10 | 2.30 | 3.02 | 3.37 |
| *p*-CA | 150 | -2.67 | 1.19 | -6.50 | 1.22 | 0.13 | 0.87 | -0.13 | 1.28 |
|  | 400 | -2.50 | 0.60 | 0.06 | 3.01 | 3.70 | 0.79 | 1.40 | 0.83 |
|  | 10000 | -2.72 | 3.31 | -4.10 | 3.93 | -1.55 | 5.21 | 6.82 | 1.49 |
| RU | 40 | -4.00 | 3.95 | 6.25 | 1.36 | -4.50 | 5.68 | 7.00 | 9.83 |
|  | 1000 | -2.48 | 1.66 | 2.90 | 11.00 | -3.06 | 4.15 | 3.36 | 4.75 |
|  | 10000 | -3.71 | 4.39 | -5.96 | 2.43 | 0.09 | 2.23 | -2.80 | 1.17 |
| SCU | 40 | -6.50 | 3.59 | 10.63 | 1.13 | 0.00 | 4.68 | 6.00 | 8.27 |
|  | 1000 | -2.90 | 3.48 | 0.30 | 4.19 | -4.00 | 4.47 | 2.80 | 6.06 |
|  | 10000 | -2.06 | 1.40 | -1.48 | 0.88 | -5.21 | 1.93 | -0.81 | 4.61 |

**4. Molecular Docking**

**Table S10** The docking total score and binding free energy ΔG of the 5 ingredients against the best docking target proteins

| **Ingredient** | **Target name**  **(Uniprot and PDB ID)** | **Total score** | **ΔG/kcal/mol** |
| --- | --- | --- | --- |
| HSYA | LTA-4H (P09960,1GW6) | 7.83 | -11.6 |
| RU | CTSB (P07858, 1CSB) | 8.35 | -9.16 |
| *p*-CA | DCK (P27707, 1P60) | 4.3 | -4.49 |
| SCU | CDK2 (P24941, 1E1V) | 9.1 | -7.83 |
| SYR | SDH (P29218, 1PL6) | 8.32 | -8.95 |

**5. Quantitative proteomics**

*5.1. Protein extraction*

Cell sample was sonicated three times on ice using a high intensity ultrasonic processor (Scientz) in lysis buffer (8 M urea, 1% Protease Inhibitor Cocktail). (Note: For PTM experiments, inhibitors were also added to the lysis buffer, e.g., 3 μM TSA and 50 mM NAM for acetylation.) The remaining debris was removed by centrifugation at 12,000 g at 4 °C for 10 min. Finally, the supernatant was collected and the protein concentration was determined with BCA kit according to the manufacturer’s instructions.

*5.2. Trypsin digestion*

For digestion, the protein solution was reduced with 5 mM dithiothreitol for 30 min at 56 °C and alkylated with 11 mM iodoacetamide for 15 min at room temperature in darkness. The protein sample was then diluted by adding 100 mM TEAB to urea concentration less than 2 M. Finally, trypsin was added at 1:50 trypsin-to-protein mass ratio for the first digestion overnight and 1:100 trypsin-to-protein mass ratio for a second 4 h-digestion.

*5.3. TMT labeling*

After trypsin digestion, peptide was desalted by Strata X C_18_ SPE column (Phenomenex) and vacuum-dried. Peptide was reconstituted in 0.5 M TEAB and processed according to the manufacturer’s protocol for TMT kit. Briefly, one unit of TMT reagent were thawed and reconstituted in acetonitrile. The peptide mixtures were then incubated for 2 h at room temperature and pooled, desalted and dried by vacuum centrifugation.

*5.4. HPLC fractionation*

The tryptic peptides were fractionated into fractions by high pH reverse-phase HPLC using Thermo Betasil C_18_ column (5 μm particles, 10 mm ID, 250 mm length). Briefly, peptides were first separated with a gradient of 8% to 32% acetonitrile (pH 9.0) over 60 min into 60 fractions. Then, the peptides were combined into 14 fractions and dried by vacuum centrifuging.

*5.5. LC-MS/MS analysis*

The tryptic peptides were dissolved in 0.1% formic acid (solvent A), directly loaded onto a home-made reversed-phase analytical column (15-cm length, 75 μm i.d.). The gradient was comprised of an increase from 6% to 23% solvent B (0.1% formic acid in 98% acetonitrile) over 26 min, 23% to 35% in 8 min and climbing to 80% in 3 min then holding at 80% for the last 3 min, all at a constant flow rate of 400 nL/min on an EASY-nLC 1000 UPLC system.

The peptides were subjected to NSI source followed by tandem mass spectrometry (MS/MS) in Q ExactiveTM Plus (Thermo) coupled online to the UPLC. The electrospray voltage applied was 2.0 kV. The *m/z* scan range was 350 to 1800 for full scan, and intact peptides were detected in the Orbitrap at a resolution of 70,000. Peptides were then selected for MS/MS using NCE setting as 28 and the fragments were detected in the Orbitrap at a resolution of 17,500. A data-dependent procedure that alternated between one MS scan followed by 20 MS/MS scans with 15.0 s dynamic exclusion. Automatic gain control (AGC) was set at 5E4. Fixed first mass was set as 100 *m/z*.

*5.6. Database search*

The resulting MS/MS data were processed using Maxquant search engine (v.1.5.2.8). Tandem mass spectra were searched against human uniprot database concatenated with reverse decoy database. Trypsin/P was specified as cleavage enzyme allowing up to 4 missing cleavages. The mass tolerance for precursor ions was set as 20 ppm in First search and 5 ppm in Main search, and the mass tolerance for fragment ions was set as 0.02 Da. Carbamidomethyl on Cys was specified as fixed modification and acetylation modification and oxidation on Met were specified as variable modifications. FDR was adjusted to < 1% and minimum score for modified peptides was set > 40.

*5.7. Bioinformatics methods*


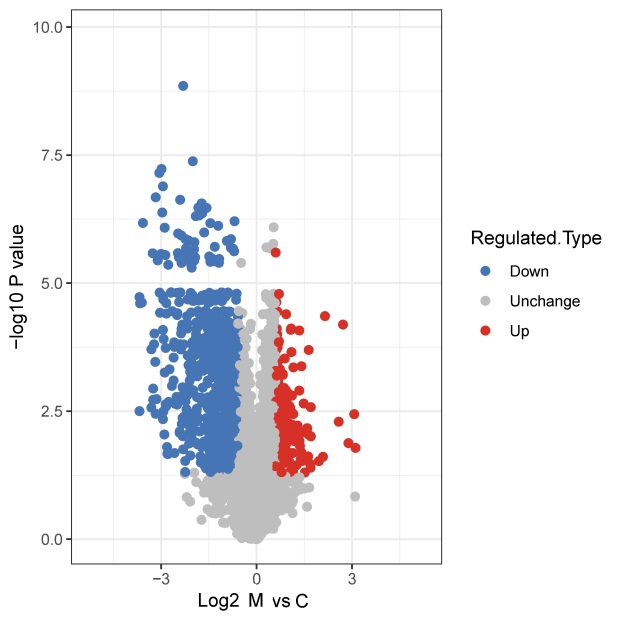

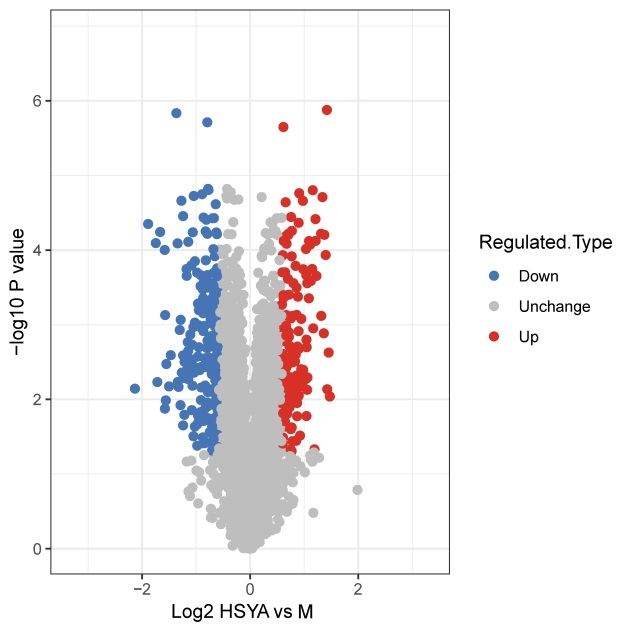

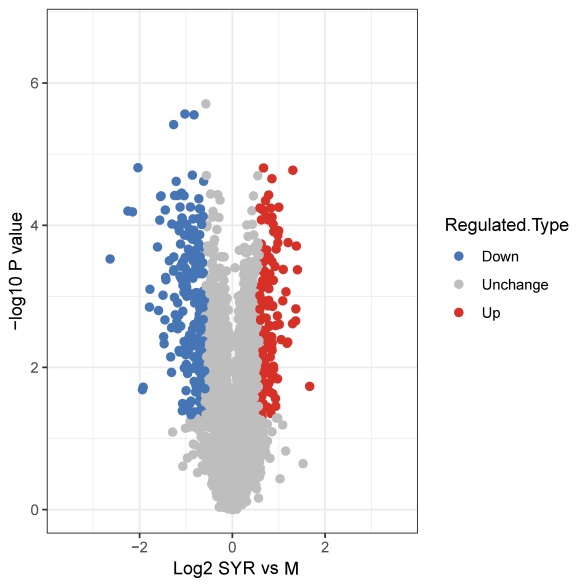


**FIGURE S4** Quantitative volcano diagrams of differentially expressed proteins.

*5.7.* *1.* *Annotation methods*

GO Annotation

The Gene Ontology, or GO, is a major bioinformatics initiative to unify the representation of gene and gene product attributes across all species. More specifically, the project aims to:

(1) Maintain and develop its controlled vocabulary of gene and gene product attributes;

(2) Annotate genes and gene products, and assimilate and disseminate annotation data;

(3) Provide tools for easy access to all aspects of the data provided by the project.

The ontology covers three domains:

(1) Cellular component: A cellular component is just that, a component of a cell, but with the proviso that it is part of some larger object; this may be an anatomical structure (e.g., rough endoplasmic reticulum or nucleus) or a gene product group (e.g., ribosome, proteasome or a protein dimer).

(2) Molecular function: Molecular function describes activities, such as catalytic or binding activities, that occur at the molecular level. GO molecular function terms represent activities rather than the entities (molecules or complexes) that perform the actions, and do not specify where or when, or in what context, the action takes place.

(3) Biological process: A biological process is series of events accomplished by one or more ordered assemblies of molecular functions. It can be difficult to distinguish between a biological process and a molecular function, but the general rule is that a process must have more than one distinct step.

Gene Ontology (GO) annotation proteome was derived from the UniProt-GOA database (http://www.ebi.ac.uk/GOA/). Firstly, converting identified protein ID to UniProt ID and then mapping to GO IDs by protein ID. If some identified proteins were not annotated by UniProt-GOA database, the InterProScan soft would be used to annotated protein’s GO functional based on protein sequence alignment method. Then proteins were classified by Gene Ontology annotation based on three categories: biological process, cellular component and molecular function. The results were shown in Figure S5.


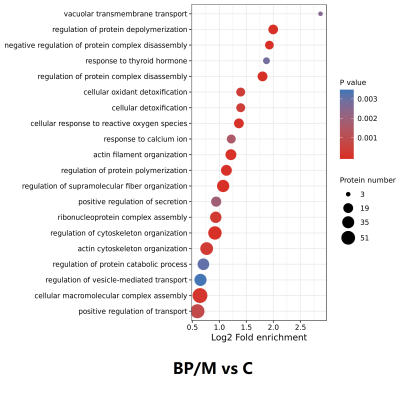

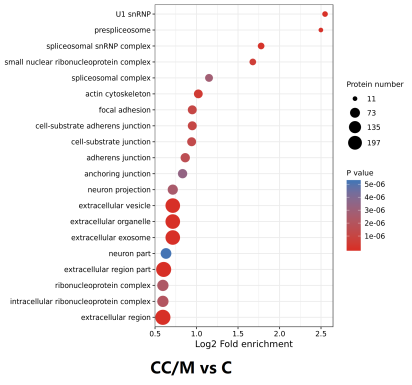

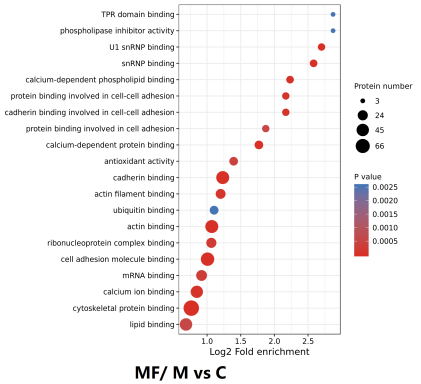


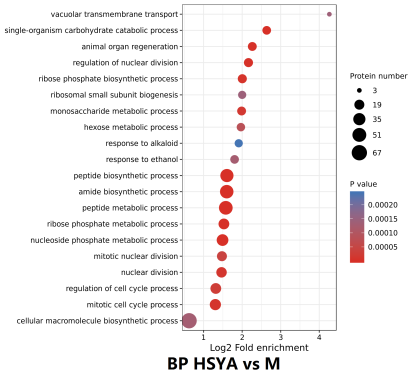

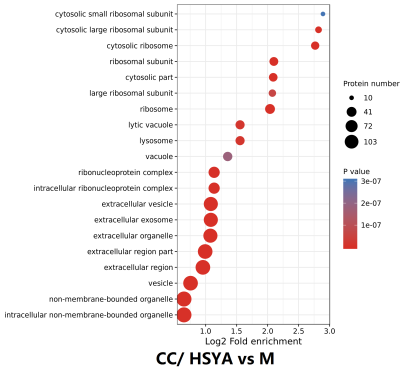

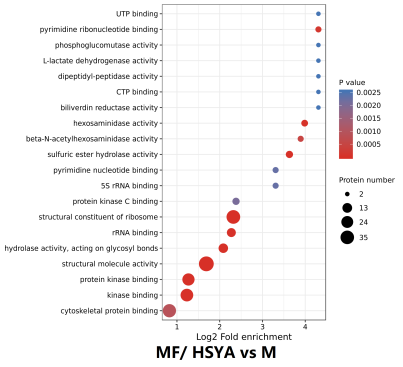


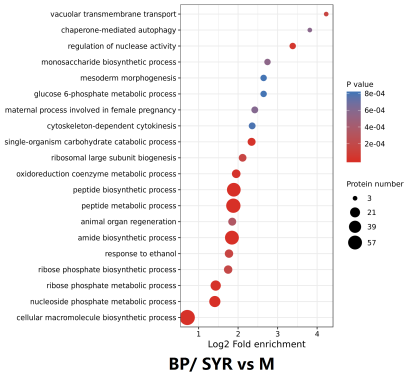

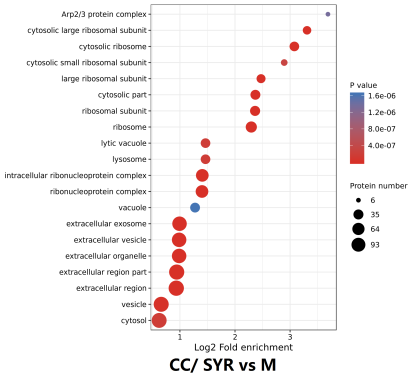

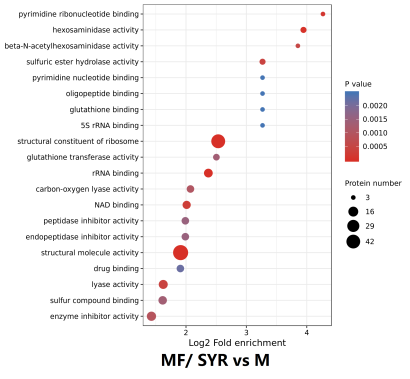


**FIGURE S5** GO enrichment analysis for the differentially expressed proteins between groups.

Subcellular Localization

The cells of eukaryotic organisms are elaborately subdivided into functionally distinct membrane bound compartments. Some major constituents of eukaryotic cells are: extracellular space, cytoplasm, nucleus, mitochondria, Golgi apparatus, endoplasmic reticulum (ER), peroxisome, vacuoles, cytoskeleton, nucleoplasm, nucleolus, nuclear matrix and ribosomes.

There, we used wolfpsort a subcellular localization predication soft to predict subcellular localization. Wolfpsort is an updated version of PSORT/PSORT II for the prediction of eukaryotic sequences. Special for protokaryon species, Subcellular localization prediction soft CELLO was used.

KEGG Pathway Annotation

KEGG connects known information on molecular interaction networks, such as pathways and complexes (the “Pathway” database), information about genes and proteins generated by genome projects (including the gene database) and information about biochemical compounds and reactions (including compound and reaction databases). These databases are different networks, known as the “protein network”, and the “chemical universe” respectively. There are efforts in progress to add to the knowledge of KEGG, including information regarding ortholog clusters in the KEGG Orthology database. KEGG Pathways mainly including: Metabolism, Genetic Information Processing, Environmental Information Processing, Cellular Processes, Rat Diseases, Drug development. Kyoto Encyclopedia of Genes and Genomes (KEGG) database was used to annotate protein pathway. Firstly, using KEGG online service tools KAAS to annotated protein’s KEGG database description. Then mapping the annotation result on the KEGG pathway database using KEGG online service tools KEGG mapper.

*5.7.2. Functional enrichment*

Enrichment of Gene Ontology analysis

Proteins were classified by GO annotation into three categories: biological process, cellular compartment and molecular function. For each category, a two-tailed Fisher’s exact test was employed to test the enrichment of the differentially expressed protein against all identified proteins. The GO with a corrected p-value < 0.05 is considered significant.

Enrichment of pathway analysis

KEGG database was used to identify enriched pathways by a two-tailed Fisher’s exact test to test the enrichment of the differentially expressed protein against all identified proteins. The pathway with a corrected p-value < 0.05 was considered significant. These pathways were classified into hierarchical categories according to the KEGG website.
